# Circulating tumor DNA in Non-Viral head and neck squamous cell Carcinoma: A systematic review and Meta-Analysis

**DOI:** 10.1016/j.oraloncology.2025.107760

**Published:** 2025-10-17

**Authors:** Vanessa Helou, Nana-Hawwa Abdul-Rahman, Suet Kee Loo, Shou-Jiang Gao, Jose P. Zevallos, Dan P. Zandberg, Matthew E. Spector, Heath D. Skinner, Robert L. Ferris, Kevin J. Contrera

**Affiliations:** a Department of Otolaryngology-Head and Neck Surgery, University of Pittsburgh, Pittsburgh, PA 15213, USA; b School of Medicine, University of Pittsburgh, Pittsburgh, PA 15213, USA; c UPMC Hillman Cancer Center, University of Pittsburgh, Pittsburgh, PA 15232, USA; d Department of Microbiology and Molecular Genetics, University of Pittsburgh, Pittsburgh, PA 15232, USA; e Department of Radiation Oncology, University of Pittsburgh, UPMC Hillman Cancer Center, Pittsburgh, PA 15232, USA; f UNC Lineberger Comprehensive Cancer Center, University of North Carolina, Chapel Hill, NC 27599, USA

**Keywords:** Head and neck squamous cell carcinoma (HNSCC), Head and neck cancer, Head and neck neoplasm, Head and neck tumor, circulating tumor DNA (ctDNA), Liquid biopsy, Precision medicine

## Abstract

Non-viral head and neck squamous cell carcinoma (HNSCC) has poor survival and high recurrence rates. Circulating tumor DNA (ctDNA) is a promising biomarker for understanding tumor biology, assessing treatment response, and monitoring disease progression. While extensively studied in virally mediated HNSCC, its role in non-viral HNSCC remains underexplored.

This systematic review and *meta*-analysis consolidates evidence on the diagnostic, prognostic, and therapeutic value of ctDNA in non-viral HNSCC. A systematic search across Medline, PubMed, Embase, and the Cochrane Library identified 1,915 records, of which 47 were included. Data extraction followed PRISMA guidelines, with overall survival (OS), progression-free survival (PFS), and recurrence-free survival (RFS), pooled as hazard ratios (HRs) with 95% confidence intervals (CIs) using a fixed-effect model.

Among 3,574 patients, the most common tumor sites were the oral cavity (35 %) and oropharynx (22 %), with the majority presenting with stage IVA/IVB disease (29 %). Pre-treatment ctDNA detection rates ranged from 50 % to 100 % (median: 83 %), while post-treatment detection rates varied between 28 % and 100 % (median: 48 %). ctDNA detected recurrence in 80 % of patients, with a median lead time of 4.6 months. ctDNA detection was significantly associated with worse OS (HR 10.26, 95 % CI 3.58–29.40; *P* < 0.0001). Residual ctDNA was strongly correlated with worse PFS (HR 7.32, 95 % CI 4.17–12.86; *P* < 0.00001) and RFS (HR 7.33, 95 % CI 2.75–19.58; *P* < 0.0001).

ctDNA holds potential for improving diagnostic accuracy, monitoring progression, and predicting survival outcomes in non-viral HNSCC. However, further large-scale studies and standardized guidelines are needed for validation and clinical implementation.

## Introduction

Oncologic outcomes for non-virally mediated head and neck squamous cell carcinoma (HNSCC) remain dismal, with minimal improvement over time.[1-3] Over 80 % of recurrences occur within the first two years of treatment, resulting in poor survival rates.[4] Non-viral HNSCC is primarily driven by environmental carcinogens, such as tobacco and alcohol, which induce complex genomic instability.[5].

Recent clinical trials, including KEYNOTE-689, have sparked growing interest in alternative methodologies for improving treatment outcomes in HNSCC.[6] However, precision treatment remains a significant challenge, underscoring the need for innovative diagnostic and prognostic tools to guide clinical decisions and enhance patient outcomes. Circulating tumor DNA (ctDNA) is an emerging non-invasive biomarker that offers insights into tumor biology, early detection, treatment monitoring, and minimal residual disease (MRD). [7-9] Non-viral ctDNA consists of DNA fragments released into the circulation from necrotic cells and actively secreted tumor cells, distinct from viral ctDNA associated with human papillomavirus (HPV) or Epstein-Barr virus (EBV). It offers potential advantages over traditional tissue biopsies, particularly for tumors in anatomically challenging locations. Although the use of ctDNA has been established in other cancers, its application in HNSCC remains underexplored and clinical guidelines for its use are still lacking. [10-12] Research on ctDNA utility in head and neck oncology has focused on virally mediated tumors, highlighting the importance of studying ctDNA in non-viral HNSCC. [13,14].

This study aimed to synthesize existing evidence on the use of ctDNA in diagnosis, prognosis, and disease management in non-viral HNSCC while identifying critical gaps in the current research.

## Methods

### Study Protocol

A systematic review and *meta*-analysis were performed of the published peer reviewed literature on ctDNA in non-viral HNSCC. We followed the Preferred Reporting Items for Systematic Reviews and Meta-Analyses (PRISMA) guidelines to report our study. [15].

### Search Strategy

A systematic search was conducted in Medline, PubMed, Embase and Cochrane library from inception to September 10, 2024. The search strategies included both keywords and medical subject headings relevant to the search terms “head and neck squamous cell carcinoma” and “circulating tumor DNA.” To ensure rigor and accuracy, we developed the search strategies with an experienced librarian (see supplement 1). We searched the databases without language or date restrictions and screened reference lists of included studies for relevant studies.

### Study selection

To be eligible for inclusion, studies needed to meet the following criteria: (i) the study population was comprised patients with HNSCC, and (ii) the study utilized a non-viral plasma ctDNA assay for diagnosis, prognosis and/or treatment response assessment. Studies exclusively focused on circulating tumor HPV DNA (ctHPV DNA) and circulating tumor EBV DNA (ctEBV DNA) were excluded, but studies including both viral and non-viral HNSCC were included. We excluded abstracts, letters to the editor, narrative reviews, and case reports.

EndNote (version 21.4) was used to retrieve articles and remove duplicate entries. [16] Two reviewers screened in duplicate and independently the titles and abstracts using Rayyan screening tool. [17] Reviewers subsequently screened the full texts in duplicate and independently. The principal investigator reviewed articles secondarily and resolved disagreements when consensus could not be reached. The reasons for excluding studies were documented.

### Data extraction

A standardized and pilot-tested data extraction form was developed. Two reviewers extracted data in duplicate from the included studies, resolving any disagreements by discussion with the principal investigator. Relevant data were extracted into an Excel spreadsheet, focusing on study characteristics, patient demographics, ctDNA assay techniques, and clinical outcomes, including overall survival (OS), progression-free survival (PFS), disease-free survival (DFS), and recurrence-free survival (RFS). Hazard ratio (HR) and 95 % confidence intervals (CI) for each outcome were extracted directly from the studies when available. We synthesized the data in narrative and tabular formats and visualized them using forest plots.

### Quality assessment

The quality of included studies was assessed in duplicate using the Newcastle-Ottawa Scale (NOS) for non-randomized trials. [18] The NOS evaluates studies on three domains: selection of study groups, comparability of groups, and assessment of outcomes. A cut-off score of 6 was used to distinguish between low and high-quality studies. By applying NOS, we ensured that the conclusions of the *meta*-analysis were based on high-quality evidence.

### Statistical analysis

All statistical analyses were performed using Cochrane Review Manager (RevMan version 8.9.0). [19] Effect estimates presented as HRs with corresponding 95 % CIs were extracted for the association between detection of ctDNA and clinical survival outcomes. To assess the degree of heterogeneity, the *I*^2^ statistic was also calculated with a value exceeding 50 % implying substantial heterogeneity. We also performed sensitivity analyses to examine the robustness of our results. Forest plots from the fixed-effect model were constructed. *Data Availability*.

The data generated in this study are available within the article and its supplementary material. Raw data may be provided upon reasonable request to the corresponding author.

## Results

### Study characteristics

Out of 1,915 retrieved citations, 47 studies met the inclusion criteria. The study selection process is depicted in PRISMA flowchart (supplement 2).

The publication years of the included studies ranged from 2001 to 2024, with a median publication year of 2022. The studies included both retrospective (n = 17, 36 %) and prospective (n = 30, 64 %) designs, with the majority originating from a single institution (n = 42, 89 %), while the remaining five studies (11 %) were multi-institutional. The United States was the most frequent country of study (n = 10, 21 %), followed by Germany and India (n = 6, 13 % each), and Canada (n = 5, 11 %). The follow-up duration ranged from 3 to 60 months, with a median of 18.5 months. The included studies involved a total of 3,574 patients with HNSCC. The range of mean age was 45–66 years (n = 11), and the range of median age was 55–71 years (n = 26). The comprehensive characteristics of the included studies, patients, ctDNA detection target, timing and methods are provided in Table 1 and supplement 3.

The most common tumor site was the oral cavity (n = 1254, 35 %) and the majority of patients had stage IVA/IVB disease (n = 1043, 29 %). Table 2 provides an overview of the distribution of tumor sites, stages, HPV status, and treatment approaches across the included studies.

### Overview of ctDNA assays and performance Metrics

Next-generation sequencing (NGS) platforms (n = 28, 60 %) and polymerase chain reaction (PCR)-based techniques (n = 21, 45 %) were used. We summarized the detailed techniques, assays, and extraction methods used in Table 3. The overall sensitivity and specificity of ctDNA assays were reported in 16 studies. The median sensitivity was 80 % (38–100 %). The median assay specificity was 99 % (60–100 %), with 12 studies exceeding 91 % specificity. The median positive predictive value (PPV) was 91 % (range 60–100 %), and the median negative predictive value (NPV) was 79 % (range 43–94 %), based on 6 studies each.

### Clinical Implications with baseline and Post-Treatment ctDNA detection

Baseline ctDNA detection rates represent the identification of ctDNA prior to any treatment. Twenty-four studies reported these rates, which ranged from 50 % to 100 %, with a median of 83 %. Out of the 24 studies,13 exceeded 80 % detection rate. Five studies reported the baseline methylated ctDNA detection rate, with a median of 59 % (range: 10–73 %). Five studies reported a median detection rate of 83 % (29–100 %) unspecified baseline ctDNA alterations. In addition, nine studies reported the baseline mutated ctDNA detection rate, with a median of 42 % (range: 26–100 %). Notable somatic mutations were *TP53, CDKN2A, PIK3CA, CASP8*, and *NOTCH1*, while DNA methylation patterns were found in *SEPT9, SHOX2, CCNA1, TIMP3, CDH8, DAPK,* and *EDNRB*. The median baseline ctDNA detection rate for *TP53* mutations was 50 % (range: 26–100 %) across five studies.

Post-treatment residual ctDNA refers to the detection of ctDNA following treatment. A total of 21 studies reported on post-treatment ctDNA detection, with detection rates varying from 28 % to 100 %, and a median of 48 %.

In 11 of these studies, ctDNA identified recurrence or progression in a median of 80 % of patients (ranging from 40 % to 100 %) prior to clinical diagnosis, with 5 studies reporting 100 % recurrence/progression detection rate. The median lead time between ctDNA detection and clinically or radiologically confirmed disease progression was 4.6 months, with a range of 1 to 27 months.

Three out of five studies found a significant difference between baseline level of ctDNA in patients with oral squamous cell carcinoma (OSCC) and controls. [20-22] The baseline ctDNA means ranged from 17.1 ± 6.9 ng/μL (mean; SD) and 109.0 ± 77.0 ng/μL. In matched healthy controls, the ctDNA means ranged from 6.0 ± 3.0 and 41.1 ± 3.1 ng/μL.

Two studies compared the baseline ctDNA levels in OSCC patients to their levels after treatment, but found no significant differences between the two groups. [22,23] In addition, Lin et al. found that patients with detectable ctDNA had a significantly higher likelihood of having OSCC, with an adjusted of odds ratio of 4.15 (95 % confidence interval [CI]: 2.16–9.21).[21].

The concordance between tumor DNA and ctDNA genetic alterations was assessed in six studies. [24-29] It ranged from 3 % and 80 %, with a median of 16 %. This highlights the variability in ctDNA detection and its relationship with tumor DNA.

### Correlation of ctDNA detection with survival outcomes

**Fig. 1.**
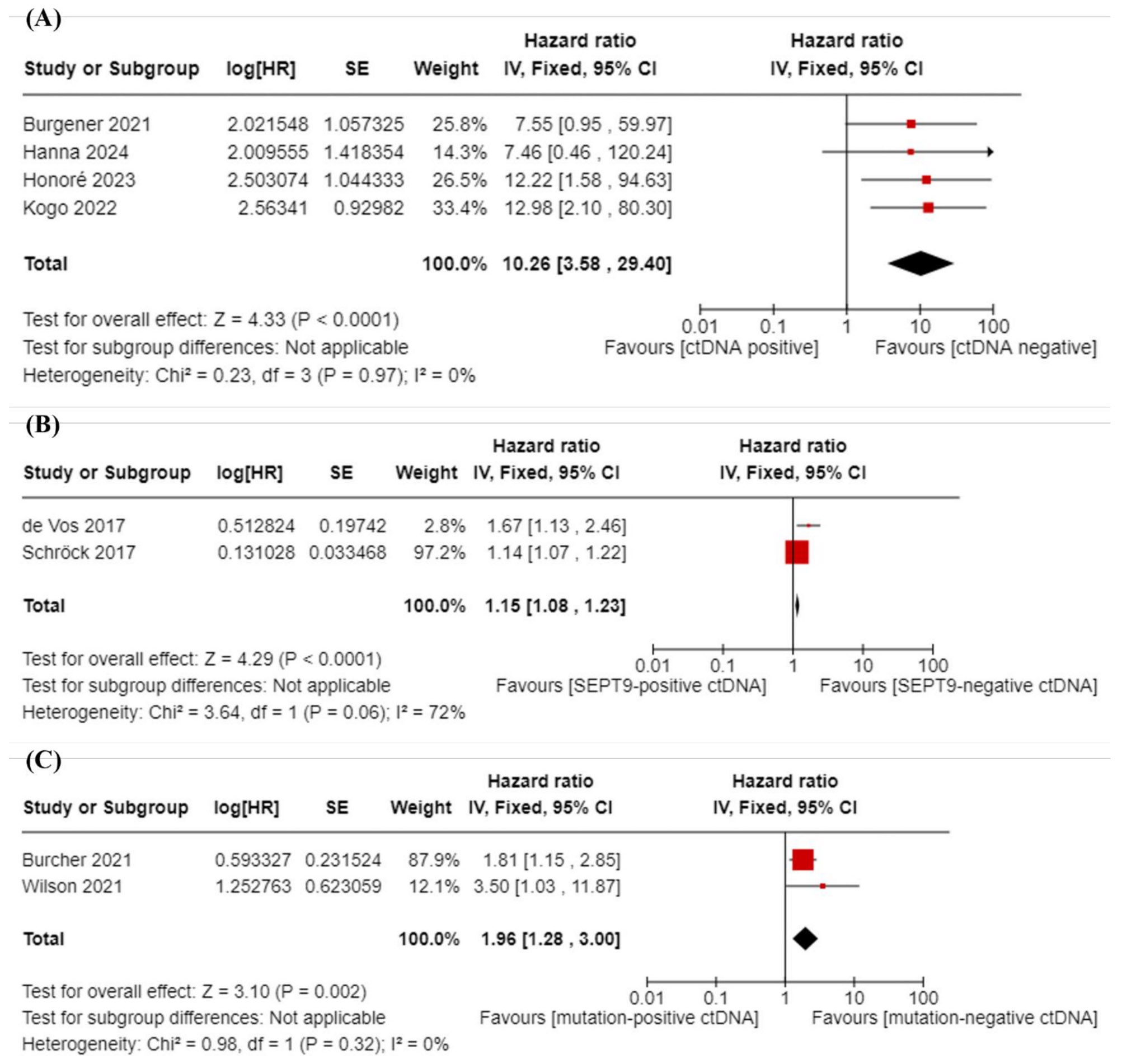
Overall survival forest plots showing the pooled hazard ratio based on **(A)** ctDNA-positive versus ctDNA-negative status, **(B)** SEPT9-positive ctDNA vs SEPT9negative ctDNA, and **(C)** mutation-positive ctDNA vs mutation-negative ctDNA. The hazard ratio for each adverse event is represented by a square, and the horizontal lines crossing the squares represent the 95% confidence interval (CI).

For OS, 13 of the 20 studies reported that the detection of ctDNA tended to correlate with worse OS, but only one study found this association to be statistically significant. In the pooled analysis (Fig. 1A), the HR from the fixed-effects model was 10.26 (95 % CI: 3.58–29.40; *P* < *0.0001*), indicating a strong association between ctDNA detection and worse OS (n = 4 studies). No heterogeneity was observed in these studies (I^2^ = 0). Fig. 1B reports a pooled HR of 1.15 (95 % CI: 1.08–1.23; *P* < *0.0001*) for the detection of *SEPT9* methylated ctDNA, suggesting that its presence is also associated with worse OS (n = 2 studies). Study heterogeneity was moderate to high (I^2^ = 72 %). Similarly, Fig. 1C shows a pooled HR of 1.96 (95 % CI: 1.28–3.00; *P* = *0.002*) for the detection of mutated ctDNA, further supporting the association between ctDNA mutations with worse OS (n = 2). No heterogeneity was observed in this analysis (I^2^ = 0).

**Fig. 2.**
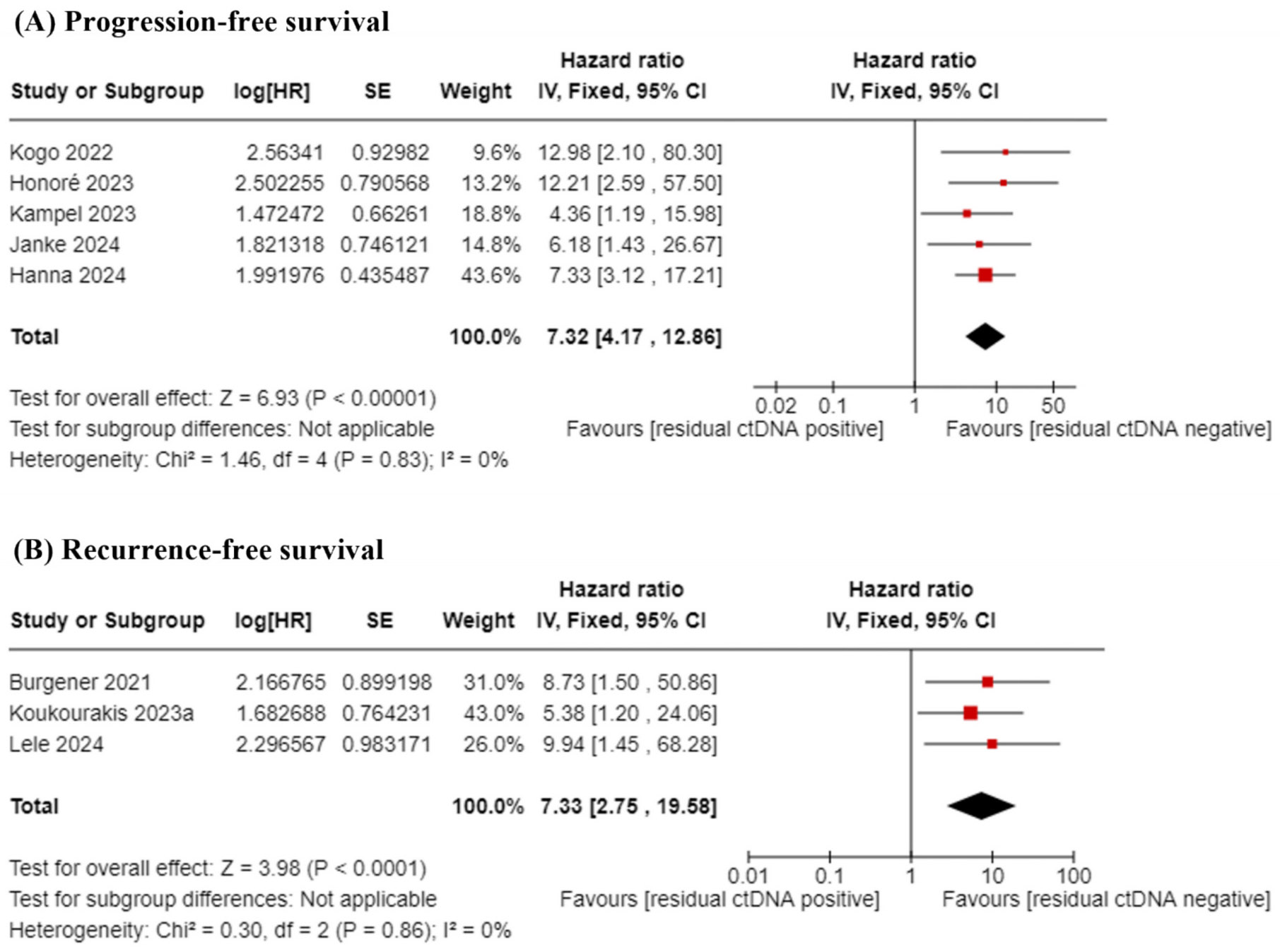
**(A)** Progression-free survival and **(B)** recurrence-free survival forest plots showing the pooled hazard ratios based on residual ctDNA-positive versus residual ctDNA-negative status. The hazard ratio for each adverse event is represented by a square, and the horizontal lines crossing the squares represent the 95% confidence interval (CI).

For PFS, eight out of 11 studies found that the presence of residual ctDNA after treatment (n = 6), *SEPT9*-positive ctDNA (n = 1) and mutations (n = 1) were associated with reduced PFS. [30-37] The remaining three studies did not identify any significant relationship, with one study reporting similar PFS between ctDNA-negative and ctDNA-positive groups. [26,38,39] The pooled HR analysis of five studies showed a strong association between residual ctDNA detection and worse PFS, with an HR of 7.32 (95 % CI: 4.17–12.86; *P* < *0.00001*) (Fig. 2A).

For RFS, the presence of residual ctDNA after treatment was identified as a significant predictor of an increased recurrence risk in six studies. The pooled HR analysis of three studies for residual ctDNA detection and RFS was 7.33 (95 % CI: 2.75–19.58; *P* < *0.0001*) (Fig. 2B). However, two studies did not find a significant relationship between ctDNA and RFS. [26,40] In addition, Chikuie et al. found that patients with detectable ctDNA had a significantly shorter median RFS of 8 months, compared to over 33 months for patients with undetectable ctDNA (*P* < *0.001*).[41].

For DFS, one study observed an association between *SEPT9*-positive ctDNA and worse DFS, with a multivariate HR of 2.72 (95 % CI: 1.30–5.68; *P* = *0.008*). [42] Another study found higher ctDNA levels to be correlated with worse DFS, with a multivariate HR of 4.43 (95 % CI: 1.21–16.18; *P* = *0.024*). [21] Khandelwal et al. reported a difference in DFS time, with ctDNA-positive patients having an average DFS of 12.3 months compared to 37.0 months for ctDNA-negative patients. [43].

## Discussion

To the best of our knowledge, this is the most comprehensive systematic review and *meta*-analysis to date examining the role of ctDNA in non-viral HNSCC. While a recent *meta*-analysis of eight studies has focused on survival outcomes in HPV-negative HNSCC, [44] our study analyzes data from 47 studies, offering a broader perspective on ctDNA’s utility in diagnosis, disease progression and recurrence monitoring, and survival outcomes. This expanded perspective significantly strengthens the evidence base, offering new insights into the clinical utility of ctDNA in non-viral HNSCC. These findings could serve as a foundation for the development of evidence-based clinical guidelines, advancing personalized management and improving survival outcomes for patients with non-viral HNSCC. All included studies had quality values greater than 6 on the NOS (supplement 4).

### ctDNA as a biomarker for diagnosis

The detection rates of ctDNA at baseline in non-viral HNSCC ranged from 50 to 100 % with a median of 83 %. These findings suggest that ctDNA is a potential diagnostic tool, with the ability to detect tumor-associated alterations in a significant proportion of patients. The most frequently detected mutations were in *TP53* and *CDKN2A*, which are associated with tumor progression, treatment resistance, and poor prognosis in HNSCC. [45-47] Furthermore, methylated ctDNA—such as *SEPT9* and *SHOX2*—was also detectable in a substantial number of patients, with a median detection rate of 59 %. The variation in detection rates may be attributed to differences in ctDNA extraction methods, assay platforms, and the biological characteristics of the tumors in addition to the variations of studied populations.

An important factor influencing the reliability and utility of ctDNA detection in clinical practice is the choice of assay. NGS platforms demonstrated high sensitivity (median 85 %) and specificity (median 100 %), while PCR-based assays showed moderate sensitivity (median 69 %) and specificity (median 96 %). These findings suggest that both techniques offer minimally invasive, real-time genotyping allowing continuous monitoring of genomic alterations and prognostic predictions. However, NGS may offer the advantage of detecting a broader range of genomic alterations, including DNA rearrangements and copy number changes, making them particularly valuable for comprehensive tumor profiling and personalized treatment strategies. [48] Despite these advantages, the heterogeneity in assay methodologies poses challenges for direct comparison across studies. Differences in analytical sensitivity, target coverage, and biomarker type can influence both detection rates and clinical interpretation, highlighting the need for standardized protocols and careful consideration of assay selection in clinical decision-making. supplement 5 summarizes the advantages and limitations of each ctDNA detection method.

### ctDNA as biomarker for predicting OS

Our *meta*-analysis revealed a strong association between the detection of ctDNA and poorer OS, with a pooled HR of 10.26 (95 % CI: 3.58–29.40; *P* < *0.0001*). This suggests that ctDNA detection can be an independent prognostic factor for worse survival outcomes in patients with non-viral HNSCC.

In terms of specific ctDNA alterations, we observed that *SEPT9* methylation and mutated ctDNA were correlated with worse OS, with pooled HRs of 1.15 and 1.96, respectively. These findings highlight that detecting specific ctDNA alterations, such as methylations or mutations, may provide complementary prognostic information. Notably, mutations in *CDKN2A* in HNSCC are associated with a higher tumor mutation burden, whether occurring as a single mutation or in combination with a *TP53* mutation. [49] However, the overall heterogeneity in results across studies evaluating *SEPT9* methylation suggests that further research is needed to validate specific ctDNA biomarkers.

### ctDNA as biomarker for monitoring progression and recurrence

Our analysis indicates that residual ctDNA following treatment is a potent predictor of disease progression and recurrence. Specifically, the pooled HR was 7.32 (95 % CI: 4.17–12.86; *P* < *0.00001*) for PFS and 7.33 (95 % CI: 2.75–19.58; *P* < *0.0001*) for RFS. Additionally, post-treatment ctDNA MRD was detected in 80 % of the patients who later experienced recurrence or progression. Importantly, ctDNA detection preceded clinically or radiologically confirmed disease recurrence or progression by approximately 4.6 months, with some studies reporting detection up to 27 months prior. These findings align with recent studies showing that ctDNA can detect MRD and predict relapse months before clinical or radiological signs of progression appear in breast, colon, and lung cancers. [50-53] For instance, post-treatment ctDNA detection preceded radiographic progression in 72 % of patients with localized lung cancers by approximately five months. [51] The potential of ctDNA to identify MRD post-treatment in patients with non-viral HNSCC earlier than standard clinical or radiological imaging may support its potential for guiding personalized adjuvant treatment at a time when the disease burden is lowest.

However, it is worth noting that four studies did not find a significant association between ctDNA detection and survival outcomes. [26,38-40] This variability may be attributed to differences in study designs, ctDNA assay methods, sample sizes, and population characteristics. Nevertheless, the strong association between ctDNA and adverse clinical outcomes depicted by pooled analyses underscores its potential as a reliable biomarker for monitoring disease progression and recurrence.

Our findings are consistent with those of other cancers in which ctDNA detection is linked to poorer survival outcomes. In metastatic breast and colorectal cancer, ctDNA detection was associated with worse OS, shorter PFS and DFS. [54,55].

### Limitations and future Directions

Despite the promising results of our analysis, several limitations should be considered. The variability in study designs, heterogeneity in ctDNA assays, patient populations, and clinical outcome definitions limits direct comparability and the generalizability of our findings. For instance, the included studies varied in terms of sample size and follow-up duration. While pooled analyses confirmed the robustness of the overall findings, future prospective studies with larger sample sizes and standardized ctDNA detection protocols are needed to validate the clinical utility of ctDNA in non-viral HNSCC.

Few studies compared ctDNA detection with traditional diagnostic biopsies. While the overall concordance between tumor DNA and ctDNA was generally low (median: 16 %), it varied widely from 3 % to 80 %. Other studies reported variable concordance rates in metastatic clear cell renal cell carcinoma (9 %), non-small cell lung cancer (83 %), and prostate cancer (89 %). [56-58] This discrepancy is likely due to both biological and technical factors. Tumor stages, tumor heterogeneity, variability in ctDNA shedding and temporal tumor evolution can result in differences between tumor tissue DNA and ctDNA. Technically, differences in assay sensitivity and specificity, as well as pre-analytical variables such as sample collection, handling, and processing, may further contribute to variability. Additionally, specific parameters assessed (e.g. concordance of mutations, methylations, and copy number alterations) may impact results. This emphasizes the need for continued effort for assay refinement and standardization, and careful interpretation of ctDNA findings.

Additionally, some studies have explored the correlation between ctDNA detection and imaging techniques. Silvoniemi et al. investigated the relationship between the detection of ctDNA somatic alterations and metabolic tumor burden measured by FDG-PET/CT in patients with treatment-naïve, locally advanced HNSCC. [59] They found that ctDNA alterations were associated with a high metabolic tumor burden. [59] In another study of 35 patients with recurrent/metastatic HNSCC receiving nivolumab or pembrolizumab monotherapy, 74 % showed concordance between ctDNA kinetics and imaging response based on Response Evaluation Criteria in Solid Tumors (RECIST). [38] These findings suggest the potential of ctDNA to assess treatment response in real-time, complementing RECIST-based imaging response assessment. However, more data is needed to fully evaluate its role and to directly assess the incremental value of ctDNA over standard imaging. This highlights the need for head-to-head comparisons to determine whether ctDNA provides actionable advantages in sensitivity, timing, or patient outcomes. While our study focused on the analytical and clinical performance of ctDNA assays, we also recognize that economic considerations are critical for implementation. Future studies should evaluate the cost-effectiveness of ctDNA-guided surveillance strategies compared with conventional radiologic imaging to inform clinical decision-making.

It is also worth mentioning that analysis of ctDNA is not yet a clinical standard. Barriers to widespread adoption include cost, insurance coverage, assay diversity, and lack of standardized pre-analytical and analytical protocols. [60,61] Additionally, the absence of formal training for clinicians on the use of ctDNA further complicates the integration of ctDNA into routine practice. [60] Other critical implementation challenges include navigating regulatory approval pathways and establishing reimbursement frameworks that can support equitable access to testing.

Despite ongoing research, a definitive consensus on the most reliable biomarkers and detection methods for non-viral HNSCC remains inconclusive. As ctDNA detection and sequencing technologies advance, standardization will be essential to enhance reproducibility and optimize clinical utility for both diagnosis and prognosis. To fully assess the benefits of incorporating ctDNA monitoring into routine management, large-scale longitudinal studies that evaluate patients before and after treatment are necessary. Additionally, the integration of plasma-based ctDNA with proximal liquid biopsy sources, such as surgical drain fluid and saliva, presents a promising avenue for improving disease monitoring and should be further explored. [9].

## Conclusions

This systematic review and *meta*-analysis of 47 studies involving over 3,500 patients provides strong evidence supporting ctDNA as a biomarker for diagnosis, prognosis, and surveillance in non-viral HNSCC. ctDNA detection was significantly associated with poorer OS, PFS, and RFS. While clinical adoption of liquid biopsy remains in early stages, these findings underscore the potential of ctDNA to inform evidence-based clinical guidelines and advance personalized management of non-viral HNSCC.

## Supplementary Material

1

2

3

4

5

## Figures and Tables

**Table 1:** Overview of the included studies: patient characteristics, ctDNA detection methods, and assay performance.

Study	Cohort size^†^	Median age (years)	Tumor site	ctDNA detection method	Initial diagnosis (yes/no)	Minimal residual disease detection (yes/no)	ctDNA assay details (sensitivity and specificity, if mentioned)
Burcher et al. 2021	170; 61 HPV-negative, 61 HPV-positive SCC	60	Oral cavity, oropharynx, larynx, hypopharynx, nasopharynx, sino-nasal, CUP	NGS	Yes	Yes	Targeted NGS using Guardant360 (Guardant Health)
Burgener et al. 2021	30; All HPV-negative SCC	NA	HNSCC (not otherwise specified)	NGS	Yes	Yes	CAPP-seq; cfMeDIP-seq
Chikuie et al. 2022	20; 5 HPV-negative, 11 HPV-positive SCC	65	Oral cavity, oropharynx, hypopharynx	NGS	Yes	Yes	Hybridization-based targeted NGS utilizing Agilent SureSelect-XT Low Input Target Enrichment, performed on the NovaSeq 6000 (Illumina) platform.
Cui et al. 2021	11	64^‡^	Oral cavity	NGS	Yes	Yes	SureSelect XT Human All Exon V5 Capture or oral cancer-specific customized gene panel was used for targeted deep sequencing, performed on the NovaSeq 6000 (Illumina) platform.
de Jesus et al. 2020	54; 32 HPV-negative, 21 HPV-positive SCC	56	Oropharynx	PCR	Yes	Yes	ddPCR Sensitivity: 73 %, Specificity: 100 %
de Vos et al. 2017	278	NA	HNSCC (not otherwise specified)	PCR	Yes	Yes	qMSP-ddPCR Sensitivity: 65 %, Specificity: 91 %
Dietrich et al. 2023	219; 32 HPV-negative, 33 HPV-positive OPSCC	64	Oral cavity, oropharynx, larynx, hypopharynx, nasal cavity, CUP	PCR	No	Yes	qMSP-ddPCR
Economopoulou et al. 2023	62; 13 HPV-positive OPSCC	65	Oral cavity, oropharynx, larynx, hypopharynx	NGS	Yes	Yes	SafeSeq
Egyud et al. 2019	8; 4 HPV-negative, 4 HPV-positive SCC	NA	Oral Cavity, hypopharynx, HNSCC (not otherwise specified)	NGS	Yes	Yes	SiMSen-Seq
Flach et al. 2022	17; All HPV-negative SCC	63	Oral cavity, oropharynx, larynx, hypopharynx	NGS	Yes	Yes	RaDaR assay (Inivata) on NovaSeq 6000 (Illumina) Sensitivity: 95 %, Specificity: 100 %
Galot et al. 2020	39; 34 HPV-negative, 5 HPV-positive SCC	NA	Oral cavity, oropharynx, larynx, hypopharynx, other sites	NGS; PCR	Yes	No	Targeted NGS (604-gene custom panel, using HiSeq4000 (Illumina)); ddPCR
Grossi et al. 2024	20; 13 HPV-negative, 7 HPV-positive SCC	NA	Oral cavity, oropharynx, larynx, hypopharynx	PCR	Yes	Yes	qMSP-ddPCR
Hanna et al. 2024	116; 44 HPV-negative, 21 HPV-positive SCC	65	Oral cavity, oropharynx, larynx, CUP, other sites	NGS	Yes	Yes	Targeted NGS using Signatera platform (Natera) Sensitivity: 93 %, Specificity: 100 %
Hilke et al. 2020	20; 17 HPV-negative, 3 HPV-positive SCC	NA	Oral cavity, oropharynx, hypopharynx	NGS (pre-and post-treatment)	Yes	Yes	Deep Sequencing
Honoré et al. 2023 [38]	53; 12 HPV-negative, 17 HPV-positive OPSCC	64	Oral cavity, oropharynx, larynx, hypopharynx, CUP	NGS	Yes	Yes	26-gene NGS panel Sensitivity: 82 %, Specificity: 79 %
Honoré et al. 2023 (2) [31]	44; 10 HPV-negative, 7 HPV-positive SCC	69	Oral cavity, oropharynx, larynx, hypopharynx, CUP	NGS	Yes	Yes	Not specified
Huang et al. 2023	148	68	Larynx	PCR	Yes	No	qPCR Sensitivity: 44 %, Specificity: 84 %
Husain et al. 2020	25	NA	Oral cavity	PCR	Yes	Yes	qPCR: CF X96 Real-Time PCR system (Bio-Rad Laboratories)
Janke et al. 2024	16; 2 HPV-negative, 1 HPV-positive SCC	59	Oral cavity, oropharynx, hypopharynx, nasopharynx, nasal cavity, sino-nasal, skull base	NGS	Yes	Yes	Low-coverage whole-genome sequencing using NovaSeq 6000 (Illumina) platform
Kakimoto et al.2008	20	62^‡^	Oral cavity	PCR	Yes	Yes	qPCR: analysis of loss of heterozygosity
Kampel et al. 2023	70; All HPV-negative SCC	65^‡^	Oral cavity, oropharynx, larynx	NGS	Yes	No	Targeted NGS using Ion Torrent platform (Life Technologies)
Khandelwal et al. 2020	22; 11 HPV-negative, 11 HPV-positive SCC	55	Oropharynx	PCR	Yes	No	qPCR Sensitivity: 100 %, Specificity: 95 %
Kogo et al. 2022	26; 22 HPV-negative, 4 HPV-positive SCC	71	Oral cavity, oropharynx, larynx, hypopharynx, external auditory canal	PCR	Yes	Yes	ddPCR
Koukourakis et al. 2023 [39]	47	66	Oral cavity, oropharynx, larynx, hypopharynx, nasopharynx, other sites (parotid and neck)	NGS	Yes	Yes	Oncomine Pan-cancer cell-free assay (Thermo Fisher Scientific)
Koukourakis et al. 2023 (2) [35]	38	64	Oral cavity, oropharynx, larynx, hypopharynx, nasopharynx, other sites (parotid and neck)	Fluorimetry	Yes	Yes	Qubit fluorometer and the Qubit 1X dsDNA HS (Thermo Fisher Scientific)
Kumari et al. 2022	68	47^‡^	Oral cavity	PCR	Yes	No	qPCR: SYBR Green real-time PCR Sensitivity: 55 %, Specificity: 60 %
Kumari et al. 2023	56; 32 HPV-negative, 7 HPV-positive SCC	55	Oropharynx	PCR	Yes	Yes	qPCR Sensitivity: 85 %, Specificity: 100 %
Lele et al. 2024	29; 7 HPV-positive SCC	65	Oral cavity, oropharynx, larynx, hypopharynx	NGS	No	Yes	Personalized tumor-informed multiplex PCR–based NGS (Signatera platform (Natera)) Sensitivity: 78 %, Specificity: 100 %
Lin et al. 2018	121	61^‡^	Oral cavity	Spectrophotometry	Yes	Yes	cfDNA quantification using TapeStation 2200 (Agilent Technology)
McKelvey et al. 2024	300	64^‡^	HNSCC (not otherwise specified)	NGS; PCR	Yes	No	NGS NOS; ddPCR
Mydlarz et al. 2016	100	58^‡^	Oral cavity, oropharynx, larynx, hypopharynx, CUP	PCR	Yes	No	qMSP-ddPCR
Nakagaki et al. 2018	80; 48 HPV-negative, 32 HPV-positive SCC	67	Oral cavity	NGS; PCR	Yes	Yes	Targeted NGS using Ion Torrent platform (Thermo Fisher Scientific); qPCR
Nunes et al. 2001	91	NA	Oral cavity, oropharynx, larynx, hypopharynx, other sites (pharynx)	PCR; Spectrophotometry	Yes	No	qPCR: analysis of loss of heterozygosity; Spectrophotometer (Spectronic-Genesys 2)
Oliva et al. 2021	10; 9 HPV-negative, 1 HPV-positive SCC	59	Oral cavity	NGS	Yes	Yes	Personalized, tumor-informed multiplex PCR–based ctDNA NGS using Signatera platform (Natera)
Payne et al. 2024	9	64^‡^	Oral cavity, oropharynx	NGS	Yes	Yes	Targeted NGS using NextSeq platform (Illumina)
Perdomo et al. 2017	73; All HPV-negative SCC	NA	Oral cavity, oropharynx, larynx, hypopharynx	NGS; PCR	Yes	No	Targeted NGS using Ion Torrent Proton Sequencer; PCR
Porter et al. 2020	60; 9 HPV-negative, 15 HPV-positive SCC	63	Oral cavity, oropharynx, larynx, hypopharynx, nasopharynx, CUP, other sites (salivary gland, thyroid)	NGS	Yes	Yes	Targeted NGS using Guardant360 (Guardant Health) Sensitivity: 85 %, Specificity: 100 %
Sanz-Garcia et al. 2024	32; 15 HPV-negative, 17 HPV-positive SCC	63	Oral cavity, oropharynx, larynx, hypopharynx	NGS; PCR	Yes	Yes	RaDaR assay (Inivata); CAPP-seq; ddPCR
Schröck et al. 2017	425	61	Oral cavity, oropharynx, larynx, hypopharynx, nasopharynx, CUP, other sites (facial skin)	PCR	Yes	Yes	qPCR: SHOX2/SEPT9 methylation Sensitivity: 59 %, Specificity: 96 %
Schwaederle et al. 2017	25	62	HNSCC (not otherwise specified)	NGS	Yes	Yes	Targeted NGS using Guardant360 (Guardant Health) Sensitivity: 85 %, Specificity: 100 %
Shukla et al. 2013	300	53^‡^	Oral cavity	Spectrophotometry	Yes	Yes	NanoDrop-1000 spectrophotometer (Thermo Fisher Scientific)
Silvoniemi et al.2023	26; 18 HPV-negative, 7 HPV-positive SCC	66^‡^	Oral cavity, oropharynx,larynx, hypopharynx	NGS	Yes	No	Targeted NGS using FoundationOne Liquid (Foundation Medicine)
Singh et al. 2024	35	45^‡^	Oral cavity, oropharynx, other sites	Spectrophotometry	Yes	No	Ultraviolet spectrophotometry Sensitivity: 43 %, Specificity: 71 %
Taylor et al. 2023	53; 37 HPV-negative, 15 HPV-positive SCC	62	Oral cavity, oropharynx, larynx, hypopharynx, nasal cavity	NGS	Yes	Yes	CAPP-seq
van Ginkel et al. 2017	6; All HPV-negative SCC	61	Oropharynx	NGS; PCR	Yes	No	Targeted NGS using Ion Torrent Platform (Thermo Fisher Scientific); QX200 ddPCR system (Bio-Rad Laboratories).
Verma et al. 2020	27	NA	Oral cavity, oropharynx, larynx	PCR	Yes	Yes	qPCR: SYBR Green real-time PCR Sensitivity: 100 %, Specificity: 100 %
Wilson et al. 2021	75; 33 HPV-negative, 20 HPV-positive SCC	60	Oral cavity, oropharynx, larynx, hypopharynx, nasopharynx, sino-nasal	NGS	Yes	Yes	Targeted NGS using Guardant360 (Guardant Health) Sensitivity: 38 %, Specificity: 98 %

**Abbreviations**: HNSCC: head and neck squamous cell carcinoma; OSCC: Oral Cavity Squamous cell carcinoma; OPSCC: Oropharyngeal Squamous Cell Carcinoma; LA HNSCC: Locoregionally Advanced HNSCC; R/M HNSCC: Recurrent/Metastatic HNSCC; CUP: Cancer Of Unknown Primary; MRD: Minimal Residual Disease; ctDNA: Circulating Tumor DNA; cfDNA: Cell-Free DNA; tDNA: Tumor DNA; HPV: Human Papillomavirus; NGS: Next-Generation Sequencing; PCR: Polymerase Chain Reaction; CAPP-seq: CAncer Personalized Profiling by deep sequencing; SiMSen-Seq**:** Sensitive mutation detection using Sequencing; SafeSeq: Safe Sequencing; cfMeDIP-seq: cell-free Methylated DNA Immuno-Precipitation sequencing; NextSeq: Next Sequencing; HiSeq: High Sequencing; ddPCR: droplet digital PCR; qMSP-ddPCR: quantitative Methylation Specific PCR.

† In some studies, the total sample size does not match the number of HPV status cases, as HPV status was not specified, or the remaining sample was matched healthy controls.

‡ Mean age is reported instead of median.

**Table 2:** Summary of the distribution of tumor sites, cancer stages, HPV status, and treatment modalities in patients with HNSCC (N = 3,574).

Variable	n (%)
**Tumor site**^§^ (N = 3,585)	
Oral cavity	1254 (35.0)
Oropharynx	797 (22.2)
Larynx	546 (15.2)
Hypopharynx	162 (4.5)
Nasopharynx	33 (0.9)
Nasal cavity	11 (0.3)
Sino-nasal	11 (0.3)
Unknown primary	37 (1.0)
Others^‡^	85 (2.4)
Not specified	649 (18.1)
**Stage** (N = 3,574)	
Early (stages I-II)	835 (23.4)
Stage III	612 (17.1)
Stage IVA/IVB	1043 (29.2)
Recurrent/Metastatic	136 (3.8)
Not specified	948 (26.5)
**HPV status** (N = 3,574)	
Negative	696 (19.5)
Positive	340 (9.5)
Not specified	2538 (71.0)
**Treatment** (N = 47 studies)	
Surgery	29 (61.7)
Radiotherapy	14 (29.8)
Chemotherapy	9 (19.1)
Chemoradiotherapy	27 (57.4)
Immunotherapy	10 (21.3)
Targeted therapy	4 (8.5)
Not specified	3 (6.4)

§ Four studies report overlapping tumor sites, resulting in a total number of tumor sites greater than the number of cases.

‡ Other sites include thyroid, salivary gland cancer, skull base, external auditory canal, parotid, neck, paranasal sinus, and facial skin.

**Table 3:** Summary of the ctDNA detection methods, assays, and extraction kits used (N = 47).

ctDNA Detection Method	n (%)
**NGS Platforms** (N = 28, 59.6 %)^◊^	
Guardant360	4 (14.3)
NovaSeq 6000	4 (14.3)
Ion Torrent PGM	4 (14.3)
Signatera	3 (10.7)
CAPP-seq	3 (10.7)
RaDaR	2 (7.1)
Oncomine Pan-cancer	1 (3.6)
FoundationOne	1 (3.6)
SiMSen-Seq	1 (3.6)
SafeSeq	1 (3.6)
cfMeDIP-seq	1 (3.6)
NextSeq	1 (3.6)
HiSeq 4000	1 (3.6)
NGS not otherwise specified	4 (14.3)
**PCR Techniques** (N = 21, 44.7 %)	
Quantitative PCR	11 (52.4)
Digital PCR	6 (28.6)
Methylation-specific PCR	4 (19.0)
**Spectrophotometry**	5 (10.6)
**DNA extraction kit** (N = 24, 51.0 %)	
QIAamp Circulating Nucleic Acid Kit	16 (34.0)
MagMAX Cell-Free DNA Kit	3 (6.4)
ChargeSwitch gDNA Serum Kit	2 (4.3)
Maxwell RSC Large Volume cfDNA Kit	2 (4.3)
GlassMax DNA Isolation Cartridge System	1 (4.2)
Not specified	23 (48.9)

**Abbreviations:** NGS: Next-Generation Sequencing; PCR: Polymerase Chain Reaction; CAPP-seq: Cancer Personalized Profiling by deep Sequencing; Ion Torrent PGM: Ion Torrent Personal Genome Machine; SiMSen-Seq**:** Sensitive mutation detection using Sequencing; SafeSeq: Safe Sequencing System; cfMeDIP-seq: cell-free Methylated DNA Immuno-Precipitation and high-throughput sequencing; NextSeq: Next Sequencing; HiSeq: High Sequencing.

◊ Three studies used two NGS platforms.

## Data Availability

Data will be made available on request.

## Appendix A. Supplementary data

Supplementary data to this article can be found online at https://doi.org/10.1016/j.oraloncology.2025.107760.

## References

[1] Menezes FS, Fernandes GA, Antunes JLF, Villa LL, Toporcov TN. Global incidence trends in head and neck cancer for HPV-related and -unrelated subsites: a systematic review of population-based studies. Oral Oncol 2021;115:105177. 10.1016/j.oraloncology.2020.105177. 33561611

[2] Du E, Mazul AL, Farquhar D, Long-term Survival in Head and Neck Cancer: Impact of Site, stage, smoking, and Human Papillomavirus Status. Laryngoscope 2019;129(11):2506–13. 10.1002/lary.27807. 30637762 PMC6907689

[3] Gormley M, Creaney G, Schache A, Ingarfield K, Conway DI. Reviewing the epidemiology of head and neck cancer: definitions, trends and risk factors. Br Dent J 2022;233(9):780–6. 10.1038/s41415-022-5166-x. 36369568 PMC9652141

[4] Hoffman HT, Karnell LH, Funk GF, Robinson RA, Menck HR. The National Cancer Data Base Report on Cancer of the Head and Neck. Archives of otolaryngology–head & neck surgery 1998;124(9):951–62. 10.1001/archotol.124.9.951. 9738803

[5] Cancer IAfRo. List of Classifications by Cancer Sites with Sufficient or Limited Evidence in Humans, Volumes 1 to 127. International Agency for Research on Cancer 2020. Accessed November 11, 2024. https://monographs.iarc.fr/agents-classified-by-the-iarc/.

[6] Merck Sharp & Dohme. Study of Pembrolizumab Given Prior to Surgery and in Combination With Radiotherapy Given Post-surgery for Advanced Head and Neck Squamous Cell Carcinoma (MK-3475-689). ClinicalTrials.gov Identifier NCT03765918. Updated April 16, 2024. Accessed November 07, 2024, https://clinicaltrials.gov/study/NCT03765918?id=NCT03765918&rank=1.

[7] Alix-Panabières C, Pantel K. Liquid Biopsy: from Discovery to Clinical Application. Cancer Discov 2021;11(4):858–73. 10.1158/2159-8290.cd-20-1311. 33811121

[8] Zviran A, Schulman RC, Shah M, Genome-wide cell-free DNA mutational integration enables ultra-sensitive cancer monitoring. Nat Med 2020;26(7):1114–24. 10.1038/s41591-020-0915-3. 32483360 PMC8108131

[9] Helou V, Smith JD, Harris M, Emerging Proximal Liquid Biopsy Approaches for Detecting Residual Disease and predicting Recurrence in Head and Neck Cancer: a Review and Proposal of Novel Liquid Staging. Head Neck Mar 21 2025.. 10.1002/hed.28138. PMC12068541 40114519

[10] Dasari A, Morris VK, Allegra CJ, ctDNA applications and integration in colorectal cancer: an NCI Colon and Rectal–Anal Task Forces whitepaper. Nat Rev Clin Oncol 2020;17(12):757–70. 10.1038/s41571-020-0392-0. 32632268 PMC7790747

[11] Pascual J, Attard G, Bidard FC, ESMO recommendations on the use of circulating tumour DNA assays for patients with cancer: a report from the ESMO Precision Medicine Working Group. Ann Oncol 2022;33(8):750–68. 10.1016/j.annonc.2022.05.520. 35809752

[12] Ettinger DS, Wood DE, Aisner DL, Non-Small Cell Lung Cancer, Version 3.2022, NCCN Clinical Practice guidelines in Oncology. J Natl Compr Canc Netw 2022;20(5):497. 10.6004/jnccn.2022.0025. 35545176

[13] Lang Kuhs KA, Brenner JC, Holsinger FC, Rettig EM. Circulating Tumor HPV DNA for Surveillance of HPV-Positive Oropharyngeal Squamous Cell Carcinoma: a Narrative Review. JAMA Oncol 2023;9(12):1716–24. 10.1001/jamaoncol.2023.4042. 37824111 PMC12011137

[14] Chan ATC, Hui EP, Ngan RKC, Analysis of Plasma Epstein-Barr Virus DNA in Nasopharyngeal Cancer After Chemoradiation to Identify High-Risk Patients for Adjuvant Chemotherapy: A Randomized Controlled Trial. J Clin Oncol. Jul 10 2018:Jco2018777847. doi:10.1200/jco.2018.77.7847. 29989858

[15] Page MJ, McKenzie JE, Bossuyt PM, The PRISMA 2020 statement: an updated guideline for reporting systematic reviews. Syst Rev 2021;10(1):89. 10.1186/s13643-021-01626-4. 33781348 PMC8008539

[16] Clarivate 2013.

[17] Ouzzani M, Hammady H, Fedorowicz Z, Elmagarmid A. Rayyan-a web and mobile app for systematic reviews. Syst Rev. Dec 5 2016;5(1):210. doi:10.1186/s13643-016-0384-4. 27919275 PMC5139140

[18] Wells GA SB, O’Connell D, Peterson J, Welch V, Losos M The Newcastle–Ottawa Scale (NOS) for assessing the quality of nonrandomised studies in meta-analyses. Accessed November 5, 2024. https://www.ohri.ca/programs/clinical_epidemiology/oxford.asp.

[19] The Cochrane Collaboration: Review Manager (RevMan). Version 8.9.0 The Cochrane Collaboration; 2024. https://community.cochrane.org/help/tools-and-software/revman.

[20] Kumari S, Mishra S, Husain N, Comparison of circulating DNA in malignant neoplasia from diverse locations: investigating a diagnostic role. Indian J Pathol Microbiol 2022;65(1):93–9. 10.4103/IJPM.IJPM_474_20. 35074971

[21] Lin L-H, Chang K-W, Kao S-Y, Cheng H-W, Liu C-J. Increased Plasma Circulating Cell-Free DNA could Be a potential Marker for Oral Cancer. Int J Mol Sci 2018;19 (11):3303. 10.3390/ijms19113303. 30352977 PMC6274798

[22] Verma T, Kumari S, Mishra S, Circulating free DNA as a marker of response to chemoradiation in locally advanced head and neck squamous cell carcinoma. Indian J Pathol Microbiol 2020;63(4):521–6. 10.4103/ijpm.ijpm_28_20. 33154299

[23] Shukla D, Kale AD, Hallikerimath S, Yerramalla V, Subbiah V. Can Quantifying Free-Circulating DNA Be a Diagnostic and Prognostic Marker in Oral Epithelial Dysplasia and Oral Squamous Cell Carcinoma? J Oral Maxillofac Surg 2013;71(2):414–8. 10.1016/j.joms.2012.04.039. 22749518

[24] Cui Y, Kim HS, Cho ES, Longitudinal detection of somatic mutations in saliva and plasma for the surveillance of oral squamous cell carcinomas. PLoS One 2021; 16(9):e0256979. 10.1371/journal.pone.0256979. 34478472 PMC8415592

[25] de Jesus LM, Dos Reis MB, Carvalho RS, Feasibility of methylated ctDNA detection in plasma samples of oropharyngeal squamous cell carcinoma patients. Head Neck Nov 2020;42(11):3307–15. 10.1002/hed.26385. 32687251

[26] Economopoulou P, Spathis A, Kotsantis I, Next-generation sequencing (NGS) profiling of matched tumor and circulating tumor DNA (ctDNA) in head and neck squamous cell carcinoma (HNSCC). Oral Oncol Apr 2023;139:106358. 10.1016/j.oraloncology.2023.106358. 36871349

[27] Galot R, van Marcke C, Helaers R, Liquid biopsy for mutational profiling of locoregional recurrent and/or metastatic head and neck squamous cell carcinoma. Article Oral Oncology 2020;104. 10.1016/j.oraloncology.2020.104631. 32169746

[28] Perdomo S, Avogbe PH, Foll M, Circulating tumor DNA detection in head and neck cancer: Evaluation of two different detection approaches. Article Oncotarget 2017;8(42):72621–32. 10.18632/ONCOTARGET.20004. 29069814 PMC5641157

[29] Wilson HL, D’Agostino RB Jr, Meegalla N, The Prognostic and Therapeutic Value of the Mutational Profile of Blood and Tumor Tissue in Head and Neck Squamous Cell Carcinoma. Oncologist Feb 2021;26(2):e279–89. 10.1002/onco.13573. 33098199 PMC7873320

[30] Hanna GJ, Dennis MJ, Scarfo N, Personalized ctDNA for Monitoring Disease Status in Head and Neck Squamous Cell Carcinoma. Clinical Cancer Research. 30 (15):3329–3336. doi:10.1158/1078-0432.CCR-24-0590. PMC11292193 38824449

[31] Honoré N, van Marcke C, Galot R, Tumor-agnostic plasma assay for circulating tumor DNA detects minimal residual disease and predicts outcome in locally advanced squamous cell carcinoma of the head and neck. Ann Oncol Dec 2023;34(12):1175–86. 10.1016/j.annonc.2023.09.3102. 37879442

[32] Janke F, Stritzke F, Dvornikovich K, Early circulating tumor DNA changes predict outcomes in head and neck cancer patients under re-radiotherapy. Int J Cancer. Aug 30 2024;doi:10.1002/ijc.35152. PMC11661516 39212345

[33] Kampel L, Feldstein S, Tsuriel S, Mutated TP53 in Circulating Tumor DNA as a Risk Level Biomarker in Head and Neck Squamous Cell Carcinoma patients. Biomolecules 2023;13(9):20. 10.3390/biom13091418. PMC10527516 37759818

[34] Kogo R, Manako T, Iwaya T, Individualized circulating tumor DNA monitoring in head and neck squamous cell carcinoma. Cancer Med Nov 2022;11 (21):3960–8. 10.1002/cam4.4726. 35352507 PMC9636504

[35] Koukourakis MI, Xanthopoulou E, Koukourakis IM, Next-Generation Sequencing Analysis of Mutations in Circulating Tumor DNA from the Plasma of patients with Head–Neck Cancer Undergoing Chemo-Radiotherapy using a PanCancer Cell-Free Assay. Article Current Oncology 2023;30(10):8902–15. 10.3390/curroncol30100643. 37887543 PMC10604986

[36] Schröck A, Leisse A, de Vos L, Free-Circulating Methylated DNA in Blood for Diagnosis, Staging, Prognosis, and monitoring of Head and Neck Squamous Cell Carcinoma patients: an Observational prospective Cohort Study. Clin Chem Jul 2017;63(7):1288–96. 10.1373/clinchem.2016.270207. 28515105

[37] Taylor K, Zou J, Burgener J, Circulating tumor DNA kinetics in recurrent/metastatic head & neck squamous cell cancer (R/M HNSCC) patients. Conference Abstract Annals of Oncology 2021;32:S796–7. 10.1016/j.annonc.2021.08.1296.

[38] Honoré N, van der Elst A, Dietz A, Tumour-agnostic plasma assay for circulating tumour DNA predicts outcome in recurrent and/or metastatic squamous cell carcinoma of the head and neck treated with a PD-1 inhibitor. Eur J Cancer 1990;2023(195):113372. 10.1016/j.ejca.2023.113372. 37913682

[39] Koukourakis MI, Xanthopoulou E, Koukourakis IM, Circulating Plasma Cell-free DNA (cfDNA) as a Predictive Biomarker for Radiotherapy: results from a prospective Trial in Head and Neck Cancer. Cancer Diagn Progn Sep-Oct 2023;3(5): 551–7. 10.21873/cdp.10254. 37671311 PMC10475926

[40] Mydlarz WK, Hennessey PT, Wang H, Carvalho AL, Califano JA. Serum biomarkers for detection of head and neck squamous cell carcinoma. Head Neck Jan 2016;38 (1):9–14. 10.1002/hed.23842. 24995714 PMC4317379

[41] Chikuie N, Urabe Y, Ueda T, Utility of plasma circulating tumor DNA and tumor DNA profiles in head and neck squamous cell carcinoma. Sci Rep 2022;12(1):9316. 10.1038/s41598-022-13417-5. 35661138 PMC9167274

[42] Dietrich D, Weider S, de Vos L, Circulating Cell-Free SEPT9 DNA Methylation in Blood is a Biomarker for Minimal Residual Disease Detection in Head and Neck Squamous Cell Carcinoma patients. Clinical chemistry (Baltimore, Md) 2023;69(9): 1050–61. 10.1093/clinchem/hvad084. 37477541

[43] Khandelwal AR, Greer AH, Hamiter M, Comparing cell-free circulating tumor DNA mutational profiles of disease-free and nonresponders patients with oropharyngeal squamous cell carcinoma. Laryngoscope investigative otolaryngology 2020;5(5):868–78. 10.1002/lio2.447. 33134534 PMC7585239

[44] Kaorey N, Dickinson K, Agnihotram VR, Zeitouni A, Sadeghi N, Burnier JV. The role of ctDNA from liquid biopsy in predicting survival outcomes in HPV-negative head and neck cancer: a meta-analysis. Oral Oncol Feb 2025;161:107148. 10.1016/j.oraloncology.2024.107148. 39742703

[45] Lawrence MS, Sougnez C, Lichtenstein L, Comprehensive genomic characterization of head and neck squamous cell carcinomas. Nature (London) 2015;517(7536):576–82. 10.1038/nature14129. 25631445 PMC4311405

[46] Chen WS, Bindra RS, Mo A, CDKN2A Copy Number loss is an Independent Prognostic factor in HPV-Negative Head and Neck Squamous Cell Carcinoma. Front Oncol 2018;8:95. 10.3389/fonc.2018.00095. 29670856 PMC5893829

[47] Pall AH, Jakobsen KK, Grønhøj C, von Buchwald C. Circulating tumour DNA alterations as biomarkers for head and neck cancer: a systematic review. Acta Oncol Jul 2020;59(7):845–50. 10.1080/0284186x.2020.1742930. 32223478

[48] Husain H, Velculescu VE. Cancer DNA in the Circulation: the Liquid Biopsy. JAMA 2017;318(13):1272–4. 10.1001/jama.2017.12131. 28973237 PMC5819336

[49] Deneka AY, Baca Y, Serebriiskii IG, Association of TP53 and CDKN2A Mutation Profile with Tumor Mutation Burden in Head and Neck Cancer. Clin Cancer Res 2022;28(9):1925–37. 10.1158/1078-0432.CCR-21-4316. 35491653 PMC9186806

[50] Peng Y, Mei W, Ma K, Zeng C. Circulating Tumor DNA and Minimal Residual Disease (MRD) in Solid Tumors: Current Horizons and Future Perspectives. Front Oncol 2021;11:763790. 10.3389/fonc.2021.763790. 34868984 PMC8637327

[51] Chaudhuri AA, Chabon JJ, Lovejoy AF, Early Detection of Molecular Residual Disease in Localized Lung Cancer by Circulating Tumor DNA Profiling. Cancer Discov 2017;7(12):1394–403. 10.1158/2159-8290.cd-17-0716. 28899864 PMC5895851

[52] Tie J, Wang Y, Tomasetti C, Circulating tumor DNA analysis detects minimal residual disease and predicts recurrence in patients with stage II colon cancer. Sci Transl Med 2016;8(346):346ra92. 10.1126/scitranslmed.aaf6219. PMC5346159 27384348

[53] Garcia-Murillas I, Schiavon G, Weigelt B, Mutation tracking in circulating tumor DNA predicts relapse in early breast cancer. Sci Transl Med 2015;7(302): 302ra133. 10.1126/scitranslmed.aab0021. 26311728

[54] Dickinson K, Sharma A, Agnihotram R-K-V, Circulating Tumor DNA and Survival in Metastatic Breast Cancer: a Systematic Review and Meta-Analysis. JAMA Netw Open 2024;7(9):e2431722. 10.1001/jamanetworkopen.2024.31722. 39235812 PMC11378006

[55] Callesen LB, Hamfjord J, Boysen AK, Circulating tumour DNA and its clinical utility in predicting treatment response or survival in patients with metastatic colorectal cancer: a systematic review and meta-analysis. Br J Cancer 2022;127(3): 500–13. 10.1038/s41416-022-01816-4. 35440666 PMC9345951

[56] Hahn AW, Gill DM, Maughan B, Correlation of genomic alterations assessed by next-generation sequencing (NGS) of tumor tissue DNA and circulating tumor DNA (ctDNA) in metastatic renal cell carcinoma (mRCC): potential clinical implications. Oncotarget 2017;8(20):33614–20. 10.18632/oncotarget.16833. 28431395 PMC5464894

[57] Prabhash K, Biswas B, Khurana S, CONCORDANCE: a real-world evidence study to evaluate the concordance of detecting epidermal growth factor receptor (EGFR) mutation by circulating tumor DNA versus tissue biopsy in patients with metastatic non-small cell lung cancer. Indian J Cancer 2022;59(5):11–8. 10.4103/ijc.ijc_438_21. 35343188

[58] Wyatt AW, Annala M, Aggarwal R, Concordance of Circulating Tumor DNA and Matched Metastatic Tissue Biopsy in Prostate Cancer. JNCI. J Natl Cancer Inst 2017;109(12)doi:10.1093/jnci/djx118. PMC6440274 29206995

[59] Silvoniemi A, Laine J, Aro K, Circulating Tumor DNA in Head and Neck Squamous Cell Carcinoma: Association with Metabolic Tumor Burden Determined with FDG-PET/CT. Cancers 2023;15(15):3970. 10.3390/cancers15153970. 37568786 PMC10416934

[60] Hasenleithner SO, Speicher MR. A clinician’s handbook for using ctDNA throughout the patient journey. Mol Cancer 2022;21(1):81. 10.1186/s12943-022-01551-7. 35307037 PMC8935823

[61] Merker JD, Oxnard GR, Compton C, Circulating Tumor DNA Analysis in patients with Cancer: American Society of Clinical Oncology and College of American Pathologists Joint Review. J Clin Oncol 2018;36(16):1631–41. 10.1200/JCO.2017.76.8671. 29504847

