# Supplementary material for: Circulating tumor DNA in Non-Viral head and neck squamous cell Carcinoma: A systematic review and Meta-Analysis
Source: Oral Oncol. Author manuscript; Available in PMC 2026 Jun 25. (PMC13299305; doi:10.1016/j.oraloncology.2025.107760)
Supplement: 1 [file NIHMS2186261-supplement-1.docx]

**Supplement 1.** Search Strategies used for electronic databases

**Medline**

1 exp "Head and Neck Neoplasms"/ 364112

2 ((((((head and neck cancer*) or head) and neck tumor*) or head) and neck neoplasm*) or H&N cancer* or H&N tumor* or H&N neoplasms or oropharyngeal cancer* or laryngeal cancer* or nasopharyngeal cancer* or hypopharyngeal cancer* or sinonasal cancer* or oral cancer*).mp. 101785

3 exp "Squamous Cell Carcinoma"/ 156066

4 (((squamous cell carcinoma of the head and neck) or (head and neck squamous cell carcinoma) or SCC of head) and neck).mp. 26120

5 1 or 2 or 3 or 4 446195

6 (circulating tumor DNA or ctDNA).mp. or exp Circulating Tumor DNA/ 7272

7 5 and 6 **313**

**Embase**

1 'head and neck cancer'/exp OR 'head and neck neoplasm*' OR 'head and neck tumor*' OR 'oropharyngeal cancer*' OR 'laryngeal cancer*' OR 'nasopharyngeal cancer*' OR 'hypopharyngeal cancer*' OR 'sinonasal cancer*' OR 'oral cancer*' OR 'head and neck squamous cell carcinoma'/exp OR 'head and neck squamous cell carcinoma' OR 'HNSCC' OR 'head and neck scc' OR 'squamous cell carcinoma of head and neck' 385,938

2 'circulating tumor DNA'/exp OR 'circulating tumor 18,393

DNA' OR 'ctDNA'

3 #1 AND #2 **838**

**PubMed**

((((((("head and neck neoplasms"[MeSH Terms] OR "head and neck neoplasms"[All Fields] OR "head and neck cancer"[All Fields] OR "head and neck tumor"[All Fields] OR "head and neck"[All Fields]) AND "tumor*"[All Fields]) OR "head and neck"[All Fields]) AND "neoplasm*"[All Fields]) OR "head and neck"[All Fields]) AND "cancer*"[All Fields]) OR (("oropharynx"[MeSH Terms] OR "oropharynx"[All Fields] OR "oropharyngeal"[All Fields]) AND "cancer*"[All Fields]) OR (("larynges"[All Fields] OR "larynx"[MeSH Terms] OR "larynx"[All Fields] OR "laryngeal"[All Fields]) AND "cancer*"[All Fields]) OR (("nasopharynx"[MeSH Terms] OR "nasopharynx"[All Fields] OR "nasopharyngeal"[All Fields]) AND "cancer*"[All Fields]) OR (("hypopharynx"[MeSH Terms] OR "hypopharynx"[All Fields] OR "hypopharyngeal"[All Fields]) AND "cancer*"[All Fields]) OR ("oral"[All Fields] AND "cancer*"[All Fields]) OR ("sinonasal"[All Fields] AND "cancer*"[All Fields]) OR ("head and neck squamous cell carcinoma"[All Fields] OR "HNSCC"[All Fields] OR "head and neck SCC"[All Fields] OR "squamous cell carcinoma of head and neck"[All Fields])) AND ("circulating tumor DNA"[MeSH Terms] OR ("circulating"[All Fields] AND "tumor"[All Fields] AND "dna"[All Fields]) OR "circulating tumor DNA"[All Fields] OR ("ctdna"[All Fields] OR "ctdnas"[All Fields])) **764**

**Cochrane Library**

("head and neck neoplasms" OR "head and neck cancer" OR "head and neck tumor" OR "head and neck" OR "head and neck neoplasms" OR "head and neck cancer" OR "head and neck tumor" OR "head and neck") AND ("tumor*" OR "neoplasm*" OR "cancer*") AND ("oropharynx" OR "oropharyngeal" OR "larynx" OR "laryngeal" OR "nasopharynx" OR "nasopharyngeal" OR "hypopharynx" OR "hypopharyngeal" OR "oral" OR "sinonasal" OR "head and neck squamous cell carcinoma" OR "HNSCC" OR "head and neck SCC" OR "squamous cell carcinoma of head and neck")

AND

("circulating tumor DNA" OR "circulating tumor DNA" OR "ctdna" OR "ctdnas")

**17**
