# Supplementary figures and images for: Circulating tumor DNA in Non-Viral head and neck squamous cell Carcinoma: A systematic review and Meta-Analysis
Source: Oral Oncol. Author manuscript; Available in PMC 2026 Jun 25. (PMC13299305; doi:10.1016/j.oraloncology.2025.107760)

**
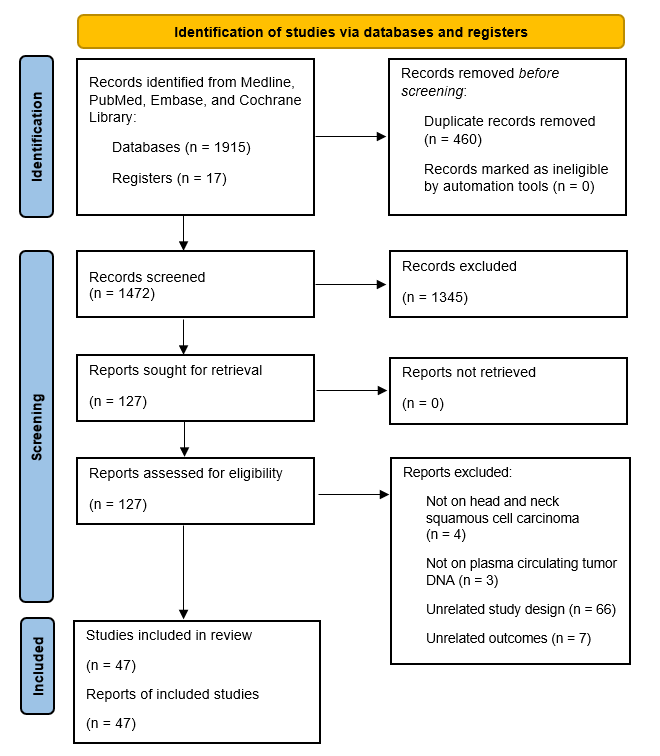
**

**Supplement 2.** PRISMA 2020 flow diagram for systematic reviews and meta-analyses.

Supplement: 2 [file NIHMS2186261-supplement-2.docx]
