# Supplementary material for: Circulating tumor DNA in Non-Viral head and neck squamous cell Carcinoma: A systematic review and Meta-Analysis
Source: Oral Oncol. Author manuscript; Available in PMC 2026 Jun 25. (PMC13299305; doi:10.1016/j.oraloncology.2025.107760)
Supplement: 3 [file NIHMS2186261-supplement-3.docx]

**Supplement 3.** Characteristics of the 47 included studies: study and patient characteristics, ctDNA detection methods, and assay performance.

| Study | Study Characteristics | | | | Patients Characteristics | | | | ctDNA Detection Details | | | |
| --- | --- | --- | --- | --- | --- | --- | --- | --- | --- | --- | --- | --- |
|  | **Study objective** | **Country** | **Study design and setting** | **Follow-up (months)** | **Tumor site** | **Cohort size** | **Median age (years)** | **Treatment** | **ctDNA biomarker detection** | **ctDNA detection method and timing** | **ctDNA assay specifics** | **Assay performance (%)** |
| Burcher et al. 2021^1^ | To determine the prevalence and prognostic value of mutated ctDNA damage repair genes in patients with HNSCC. | USA | Retrospective; single-institutional | 20.2 | Oral Cavity, Oropharynx, Larynx, Hypopharynx, Nasopharynx, Sino-Nasal, CUP | 170; 61 with HPV-negative SCC, 61 with HPV-positive SCC | 60 | Chemotherapy and/or radiation therapy | ctDNA mutations (damage repair genes); (*APC, ARID1A, APC, BRCA1, BRCA2* and  *CDK12)* | NGS  (pre-and post-treatment) | Guardant360^®^ (Guardant Health); | NA |
| Burgener et al. 2021^2^ | To determine the feasibility of tumor-naïve detection of ctDNA by simultaneously profiling mutations and methylation in locoregionally confined HPV-negative HNSCC. | Canada | Prospective; single-institutional | 41.2 | HNSCC NOS | 30; All HPV-negative SCC | NA | Surgery with or without adjuvant radiation therapy /chemoradiotherapy | ctDNA gene mutations and methylation detection; (*TP53, PIK3CA, FAT1, NOTCH1, GRIN3A,* and *MYC)* | NGS  (pre-and post-treatment) | CAPP-seq for somatic mutation detection; cfMeDIP-seq for methylation profiling; | NA |
| Chikuie et al. 2022^3^ | To identify the role of ctDNA in treatment monitoring and recurrence detection in patients with HNSCC and clarify the clinical utility of gene mutation analysis. | Japan | Prospective; single-institutional | 24 | Oral Cavity, Oropharynx, Hypopharynx | 20; 5 with HPV-negative SCC, 11 with HPV-positive SCC | 65 | Surgery, chemotherapy and/or radiation therapy | ctDNA detection; (*KMT2D, PCLO, KMT2C, TP53, SDHA*, and *NOTCH1)* | NGS  (pre-and post-treatment) | Hybridization-based targeted NGS utilizing Agilent SureSelect-XT Low Input Target Enrichment, performed on the NovaSeq 6000 (Illumina) platform. | NA |
| Cui et al. 2021^4^ | To examine the feasibility of serial liquid biopsy in detecting MRD in patients with oral cancer. | Republic of Korea | Prospective; single-institutional | 18 | Oral Cavity | 11 | 64‡ | Surgery, chemotherapy and/or radiation therapy | ctDNA mutation detection (*CASP8, TP53, CASP8, AJUBA, CDKN2A,* and *NOTCH1)* | NGS  (pre-and post-treatment) | SureSelect XT Human All Exon V5 Capture library for  WES or oral cancer-specific customized gene panel was used for targeted deep sequencing, performed on the NovaSeq 6000 (Illumina) platform. | NA |
| de Jesus et al. 2020^5^ | To evaluate methylation-based markers in plasma from OPSCC patients as emerging tools for accurate, noninvasive follow-up. | Brazil | Retrospective; single-institutional | 17.7 | Oropharynx | 54; 32 with HPV-negative SCC, 21 with HPV-positive SCC | 56 | Surgery with or without adjuvant radiation therapy /chemotherapy | ctDNA methylation detection (*CCNA1, TIMP3, CDH8,* and *DAPK*) | PCR  (pre-and post-treatment) | ddPCR | Sensitivity: 73%, Specificity: 100% |
| de Vos et al. 2017^6^ | To compare different quantification algorithms in order to improve the clinical performance of methylated cfDNA as a biomarker with the example of SEPT9 and SHOX2 in HNSCC. | Germany | Retrospective; single-institutional | NA | HNSCC NOS | 278 | NA | Surgery, chemotherapy and/or radiation therapy | ctDNA methylation detection (*SHOX2* and *SEPT9*) | PCR  (pre-and post-treatment) | qMSP-ddPCR | Sensitivity: 65%, Specificity: 91% |
| Dietrich et al. 2023^7^ | To evaluate the clinical performance of SEPT9 cfDNA methylation to detect post-surgical MRD in patients with localized or locally advanced HNSCC treated with curative intent. | Germany | Prospective; single-institutional | 27 | Oral Cavity, Oropharynx, Larynx, Hypopharynx, Nasal Cavity, CUP | 219; 32 with HPV-negative OPSCC, 33 with HPV-positive OPSCC | 64 | Surgery with or without adjuvant radiation therapy /chemoradiotherapy | ctDNA methylation detection (*SEPT9*) | PCR  (post-treatment/surgery) | qMSP-ddPCR | NA |
| Economopoulou et al. 2023^8^ | To evaluate the presence of somatic mutations in matched tumor and ctDNA samples from patients with primary HNSCC and assess the association of changes in ctDNA levels with survival. | Greece | Prospective; single-institutional | 25 | Oral Cavity, Oropharynx, Larynx, Hypopharynx | 62; 13 with HPV-positive OPSCC | 65 | Surgery or radical chemoradiotherapy | ctDNA mutation detection (*TP53, CDKN2A, HRAS*  and *PI3KCA)* | NGS  (pre-and post-treatment) | SafeSeq | NA |
| Egyud et al. 2019^9^ | To quantify patient-specific changes in ctDNA following curative therapy and to examine the potential role of ctDNA in treatment monitoring and recurrence detection in HNSCC. | USA, Sweden, Canada | Prospective; multi-institutional | 12 | Oral Cavity, Hypopharynx, HNSCC NOS | 8; 4 with HPV-negative SCC, 4 with HPV-positive SCC | NA | Surgery with or without adjuvant chemoradiation therapy | ctDNA mutation detection (including *TP53, ARID1B,*  *ATM, CDK8, FANCA,* and *RASA1*) | NGS  (pre-and post-treatment) | SiMSen-Seq | NA |
| Flach et al. 2022^10^ | To determine whether post-operative ctDNA detection can act as a biomarker for surgical tumor clearance in HPV-negative HNSCC and to evaluate the potential of personalized ctDNA analysis for early molecular-level detection of relapse prior to clinically confirmed recurrence with concomitant implications for therapy planning. | Germany | Prospective; single-institutional | 12.2 | Oral Cavity, Oropharynx, Larynx, Hypopharynx | 17; All HPV-negative SCC | 63 | Surgery with or without adjuvant radiation therapy /chemotherapy | ctDNA somatic variants detection | NGS  (pre-and post-treatment) | RaDaR^TM^ assay (Inivata) | Sensitivity: 95%, Specificity: 100% |
| Galot et al. 2020^11^ | To investigate the feasibility of detecting ctDNA in R/M HNSCC patients using a tissue-agnostic approach (without prior knowledge of somatic mutations in the solid tumor) and to assess how ctDNA reflects the mutational landscape of the tumor. | Belgium | Prospective; single-institutional | NA | Oral Cavity, Oropharynx, Larynx, Hypopharynx, Other Sites | 39; 34 with HPV-negative SCC, 5 with HPV-positive SCC | NA | Surgery with or without adjuvant radiation therapy /chemotherapy | ctDNA mutation detection (including *TP53*) | NGS; PCR  (baseline) | Targeted NGS (604-gene custom panel, Illumina HiSeq 4000); ddPCR | NA |
| Grossi et al. 2024^12^ | To evaluate DNA methylation levels of SHOX2 and SEPT9 during treatment in HNSCC using ddPCR. | Italy | Prospective; single-institutional | 12 | Oral Cavity, Oropharynx, Larynx, Hypopharynx | 20; 13 with HPV-negative SCC, 7 with HPV-positive SCC | NA | Surgery with or without adjuvant radiation therapy /chemotherapy | ctDNA methylation detection (*SHOX2* and *SEPT9*) | PCR  (pre-and post-treatment) | qMSP-ddPCR | NA |
| Hanna et al. 2024^13^ | To evaluate the performance of a custom-built, tumor-informed, commercially available ctDNA assay in predominantly HPV-negative HNSCC patients for monitoring disease status. | USA | Retrospective; single-institutional | 5.1 | Oral Cavity, Oropharynx, Larynx, CUP, Other Sites | 116; 44 with HPV-negative SCC, 21 with HPV-positive SCC | 65 | Surgery with or without adjuvant radiation therapy /chemotherapy or chemoradiotherapy alone | ctDNA detection (tumor-informed somatic variants) | NGS  (pre-and post-treatment) | Signatera™ platform (Natera) | Sensitivity: 93%, Specificity: 100% |
| Hilke et al. 2020^14^ | To evaluate the capacity of ctDNA as a treatment response biomarker in patients with HNSCC receiving radiochemotherapy. | Germany | Prospective; single-institutional | 27.1 | Oral Cavity, Oropharynx, Hypopharynx | 20; 17 with HPV-negative SCC, 3 with HPV-positive SCC | NA | Chemoradiotherapy | ctDNA mutation detection (including *TP53, NOTCH* and *PI3K*) | NGS  (pre-and post-treatment) | Deep Sequencing | NA |
| Honoré et al. 2023^15^ | To develop a tumor-agnostic plasma ctDNA assay to detect MRD in unselected LA HNSCC with the aim of predicting progression-free survival and overall survival without the need for tumor sequencing. | Belgium | Prospective; single-institutional | 31 | Oral Cavity, Oropharynx, Larynx, Hypopharynx, CUP | 53; 12 with HPV-negative OPSCC, 17 with HPV-positive OPSCC | 64 | Surgery or chemoradiotherapy or radiation therapy | ctDNA mutation detection (including *TP53, NOTCH1, EGFR* and *FAT1*) , variant allele frequency | NGS  (pre-and post-treatment) | 26-gene NGS panel | Sensitivity: 82%, Specificity: 79% |
| Honoré et al. 2023 (2)^16^ | To evaluate tumor-agnostic ctDNA plasma assay in predicting the efficacy of nivolumab or pembrolizumab monotherapy in R/M HNSCC. | Belgium | Prospective; multi-institutional | NA | Oral Cavity, Oropharynx, Larynx, Hypopharynx, CUP | 44; 10 with HPV-negative SCC, 7 with HPV-positive SCC | 69 | Surgery with or without chemoradiation therapy or chemoradiation therapy alone or radiation therapy alone or none | ctDNA mutation detection (including *TP53, SPEN, EP300, SMG1,* and *KMT2C*), variant allele frequency | NGS  (pre-and post-treatment) | Not specified | NA |
| Huang et al. 2023^17^ | To identify the diagnostic and prognostic value of cfDNA and vascular endothelial growth factor-C (VEGF-C) in laryngeal squamous cell carcinoma. | China | Prospective; single-institutional | NA | Larynx | 148 | 68 | Surgery alone | ctDNA detection levels (ALU115, ALU247, and cfDNA integrity  index) | PCR  (baseline/pre-treatment) | qPCR | Sensitivity: 44%, Specificity: 84% |
| Husain et al. 2020^18^ | To evaluate ctDNA as a marker of adequate resection after upfront surgery for OSCC and its correlation with clinical and pathologic parameters. | India | Prospective; single-institutional | NA | Oral Cavity | 25 | NA | Surgery alone | ctDNA detection levels (ALU115 and ALU247) | PCR  (pre- and post-treatment) | qPCR: CF X96 Real-Time PCR system (Bio-Rad Laboratories) | NA |
| Janke et al. 2024^19^ | To evaluate copy number variations (CNV)-based ctDNA dynamics in predicting outcome in locoregional recurrent HNSCC patients under re-radiotherapy. | Germany | Prospective; single-institutional | NA | Oral Cavity, Oropharynx, Hypopharynx, Nasopharynx, Nasal Cavity, Sino-Nasal, Skull Base | 16; 2 with HPV-negative SCC, 1 with HPV-positive SCC | 59 | Prior therapy (surgery, radiotherapy, chemotherapy), underwent re-radiotherapy | ctDNA detection levels (copy number variation) | NGS  (baseline before re-radiotherapy and post-treatment) | Low-coverage WGS using NovaSeq 6000 (Illumina) | NA |
| Kakimoto et al. 2008^20^ | To determine the feasibility of obtaining a prognosis of OSCC by microsatellite analysis of serum DNA. | Japan | Retrospective; single-institutional | 32 | Oral Cavity | 20 | 62‡ | Surgery with or without preoperative chemotherapy | ctDNA microsatellite alterations detection | PCR  (pre- and post-treatment) | qPCR: analysis of loss of heterozygosity | NA |
| Kampel et al. 2023^21^ | To assess the prognostic value and the potential translational applicability of ctDNA as a surrogate marker for high-risk disease in newly diagnosed resectable HNSCC. | Israel | Retrospective; single-institutional | 16.5 | Oral Cavity, Oropharynx, Larynx | 70; All HPV-negative SCC | 65‡ | Surgery with or without adjuvant chemotherapy or radiation therapy | ctDNA mutation detection (*TP53*) | NGS  (pre-treatment) | Targeted NGS (TP53 panel) using Ion Torrent Platform (Life Technologies) | NA |
| Khandelwal et al. 2020^22^ | To determine if somatic nonsynonymous variants could be identified in cfDNA of OPSCC patients and investigate the extent to which the mutations found in the ctDNA correlated to those altered in paired tumor tissues. | USA | Retrospective; single-institutional | 38.6 | Oropharynx | 22; 11 with HPV-negative SCC, 11 with HPV-positive SCC | 55 | Surgery with or without adjuvant chemotherapy or radiation therapy | ctDNA mutation detection (including *TP53*, *PIK3CA,* and *CDKN2A*) | PCR  (pre-treatment) | qPCR | Sensitivity: 100%, Specificity: 95% |
| Kogo et al. 2022^23^ | To evaluate the role of individualized ctDNA monitoring using dPCR in reflecting the clinical course and potential clinical applications of patients with HNSCC. | Japan | Prospective; single-institutional | 18.5 | Oral Cavity, Oropharynx, Larynx, Hypopharynx, Other Sites (External Auditory Canal) | 26; 22 with HPV-negative SCC, 4 with HPV-positive SCC | 71 | Surgery with or without chemoradiation therapy or chemoradiotherapy alone or radiation therapy alone or chemotherapy alone or induction chemotherapy alone | ctDNA mutation detection (variant allele frequency) (*TP53, PIK3CA,*  *KMT2D, CDKN2A, FAT1,*  *FBXW8, NOTCH3,* and  *CREBBP*) | PCR  (pre- and post-treatment) | ddPCR | NA |
| Koukourakis et al. 2023^24^ | To identify specific gene mutations involved in resistance to chemo-radiotherapy and also assess an eventual prognostic role of persistent or newly emerging mutations after treatment. | Greece | Prospective; single-institutional | 16 | Oral Cavity, Oropharynx, Larynx, Hypopharynx, Nasopharynx, Other Sites (Parotid and Neck) | 47 | 66 | Chemoradiation therapy | ctDNA mutation detection (including *TP53, EGFR, AR, FGFR3,*  and *FBXW3*) | NGS  (pre- and post-treatment) | Oncomine Pan-cancer cell-free assay (Thermo Fisher Scientific) | NA |
| Koukourakis et al. 2023 (2)^25^ | To evaluate the role of plasma cfDNA concentration in the outcome of radiotherapy of patients with HNSCC. | Greece | Prospective; single-institutional | 15 | Oral Cavity, Oropharynx, Larynx, Hypopharynx, Nasopharynx, Other Sites (Parotid and Neck) | 38 | 64 |  | ctDNA detection levels | Fluorimetry  (pre- and post-treatment) | Qubit fluorometer and the Qubit 1X dsDNA HS (Thermo Fisher Scientific) | NA |
| Kumari et al. 2022^26^ | To quantitatively evaluate the levels of serum cfDNA and compare difference across a spectrum of diverse malignant tumors and in healthy controls. | India | Prospective; single-institutional | NA | Oral Cavity | 68 | 47‡ | Not specified (pre-treatment) | ctDNA detection levels | PCR  (pre-treatment) | qPCR: SYBR Green real-time PCR | Sensitivity: 55%, Specificity: 60% |
| Kumari et al. 2023^27^ | To use liquid biopsy as a non-invasive biomarker for OPSCC by assessing serum cfDNA levels, methylations, and compared the results to available formalin fixed paraffin embedded tissue. | India | Prospective; single-institutional | 3 | Oropharynx | 56; 32 with HPV-negative SCC, 7 with HPV-positive SCC | 55 | Chemoradiotherapy | ctDNA detection levels, and methylation detection (*P16*, *DAPK* and *RASSF1A*) | PCR  (pre- and post-treatment) | qPCR | Sensitivity: 85%, Specificity: 100% |
| Lele et al. 2024^28^ | To assess the utility of ctDNA in the surveillance of HNC patients and help establish its role as an adjunct to PET scans in assessing response to treatment. | USA | Retrospective; single-institutional | 16 | Oral Cavity, Oropharynx, Larynx, Hypopharynx | 29; 7 with HPV-positive SCC | 65 | Surgery or chemoradiotherapy | ctDNA detection | NGS  (post-treatment) | Personalized tumor-informed multiplex PCR–based NGS (Signatera platform (Natera)) | Sensitivity: 78%, Specificity: 100% |
| Lin et al. 2018^29^ | To evaluate whether plasma cfDNA levels could be a potential non-invasive marker for OSCC. | Taiwan | Prospective; single-institutional | 28 | Oral Cavity | 121 | 61‡ | Surgery with or without adjuvant chemotherapy or radiation therapy | ctDNA detection levels | Spectrophotometry  (pre- and post-treatment) | cfDNA quantification using TapeStation 2200 (Agilent Technology) | NA |
| McKelvey et al. 2024^30^ | To assess baseline ctDNA levels across five cancer types in early- and late-stage disease from a multi-assay study. | USA | Retrospective; multi-institutional | NA | HNSCC NOS | 300 | 64‡ | Not specified (pre-treatment) | ctDNA detection levels | NGS; PCR  (pre-treatment) | NGS NOS; ddPCR | NA |
| Mydlarz et al. 2016^31^ | To investigate the potential of methylation ctDNA in the serum of pretreatment of patients with HNSCC for early detection. | USA | Retrospective; single-institutional | NA | Oral Cavity, Oropharynx, Larynx, Hypopharynx, CUP | 100 | 58‡ | Not specified (pre-treatment) | ctDNA methylation detection (*EDNRB* hypermethylation) | PCR  (pre-treatment) | qMSP-ddPCR | NA |
| Nakagaki et al. 2018^32^ | To evaluate targeted NGS as tool for mutation analysis of 50 cancer-related genes in OSCC. | Japan | Prospective; single-institutional | NA | Oral Cavity | 80; 48 with HPV-negative SCC, 32 with HPV-positive SCC | 67 | Surgery | ctDNA mutation detection (including *TP53*  *CDKN2A, PIK3CA, HRAS,* and *MET*) | NGS; PCR  (pre- and post-treatment) | Targeted NGS using Ion Torrent Platform (Thermo Fisher Scientific); qPCR | NA |
| Nunes et al. 2001^33^ | To evaluate the value of ctDNA in early diagnosis of HNSCC. | Brazil | Retrospective; single-institutional | NA | Oral Cavity, Oropharynx, Larynx, Hypopharynx, Other Sites (Pharynx NOS) | 91 | NA | Surgery | ctDNA mutation detection | PCR; Spectrophotometry  (pre-treatment) | qPCR: analysis of loss of heterozygosity | NA |
| Oliva et al. 2021^34^ | To evaluate the immune and molecular effects of preoperative Sitravatinib and Nivolumab in patients with OSCC. | Canada | Prospective; single-institutional | 21 | Oral Cavity | 10; 9 with HPV-negative SCC, 1 with HPV-positive SCC | 59 | Neoadjuvant immunotherapy, surgery with or without adjuvant radiotherapy | ctDNA mutation detection (including *TP53, FAT1,* and *NOTCH1*), variant-allele  frequency | NGS  (pre- and post-treatment) | Personalized, tumor-informed multiplex PCR–based ctDNA NGS using Signatera™ platform (Natera) | NA |
| Payne et al. 2024^35^ | To investigate if genomic spatial heterogeneity identified from sequencing of HNSCC tumors is detectable in ctDNA and to examine the efficacy of ctDNA to detect clones contributing to heterogeneity. | United Kingdom | Prospective; single-institutional | NA | Oral Cavity, Oropharynx | 9 | 64‡ | Surgery with or without adjuvant chemoradiotherapy or radiation therapy or chemoradiotherapy alone | ctDNA mutation detection (including *TP53, NOTCH1, PIK3CA,*  *KMT2D, CDKN2A, CASP8, NSD1, FAT1* and *FBXW7*) | NGS  (pre- and post-treatment) | Targeted NGS (9-gene panel, tumor-informed, Illumina NextSeq). | NA |
| Perdomo et al. 2017^36^ | To evaluate the presence of ctDNA in plasma and oral rinses in HNSCC cases at early and late stages and to determine the best approach to use ctDNA in HNSCC early detection. | South America and Argentina | Retrospective; multi-institutional | 60 | Oral Cavity, Oropharynx, Larynx, Hypopharynx | 73; All HPV-negative SCC | NA | Not specified (pre-treatment) | ctDNA mutation detection (*TP53*) | NGS; PCR  (pre-treatment) | Targeted NGS using Ion Torrent Proton Sequencer; PCR | NA |
| Porter et al. 2020^37^ | To characterize plasma cfDNA in advanced HNSCC patients, determine its ability to identify mutations, and elucidate its potential role in management. | USA | Retrospective; single-institutional | NA | Oral Cavity, Oropharynx, Larynx, Hypopharynx, Nasopharynx, CUP, Other Sites (Salivary Gland, Thyroid) | 60; 9 with HPV-negative SCC, 15 with HPV-positive SCC | 63 | Surgery with or without chemotherapy or chemoradiation therapy | ctDNA mutation detection (including *TP53*, and *PIK3A*) | NGS  (pre- and post-treatment) | Targeted NGS using Guardant360^®^ (Guardant Health) | Sensitivity: 85%, Specificity: 100% |
| Sanz-Garcia et al. 2024^38^ | To correlate the detection of ctDNA with clinical relapse at baseline, and at follow-up and to explore whether there is a preferred method and timepoint for MRD detection. | Canada | Prospective; single-institutional | 25 | Oral Cavity, Oropharynx, Larynx, Hypopharynx | 32; 15 with HPV-negative SCC, 17 with HPV-positive SCC | 63 | Surgery with or without adjuvant radiation therapy or chemoradiation or radiation therapy alone or chemoradiation therapy alone | ctDNA mutation detection and variant allele frequency | NGS; PCR  (pre- and post-treatment) | RaDaR^TM^ assay (Inivata); CAPP-seq; ddPCR | NA |
| Schröck et al. 2017^39^ | To explore the value of quantitative SEPT9 and SHOX2 methylation levels in cfDNA for the clinical management of HNSCC patients. | Germany | Prospective; single-institutional | NA | Oral Cavity, Oropharynx, Larynx, Hypopharynx, Nasopharynx, CUP, Other Sites (Facial Skin) | 425 | 61 | Surgery with or without chemotherapy  and/or radiation therapy | ctDNA methylation detection (*SHOX2* and *SEPT9*) | PCR  (pre- and post-treatment) | qPCR: SHOX2/SEPT9 methylation | Sensitivity: 59%, Specificity: 96% |
| Schwaederle et al. 2017^40^ | To investigate the genomic alterations in ctDNA from diverse cancer patients identified by NGS. | USA | Retrospective; single-institutional | NA | HNSCC NOS | 25 | 62 | Not specified | ctDNA mutation detection (including *TP53, EGFR, KRAS* and *PIK3CA*) | NGS  (pre- and post-treatment) | Targeted NGS using Guardant360^®^ (Guardant Health) | Sensitivity: 85%, Specificity: 100% |
| Shukla et al. 2013^41^ | To determine whether cfDNA can aid in the diagnosis and prognosis of oral epithelial dysplasia and OSCC. | India | Retrospective; single-institutional | 24 | Oral Cavity | 300 | 53‡ | Surgery with or without chemotherapy  and/or radiation therapy | ctDNA detection levels | Spectrophotometry  (pre- and post-treatment) | NanoDrop-1000 spectrophotometer (Thermo Fisher Scientific) | NA |
| Silvoniemi et al. 2023^42^ | To examine the association between mutational status on ctDNA and tDNA and the metabolic tumor burden observed in FDG-PET/CT imaging in treatment naïve HNSCC patients. | Finland | Prospective; multi-institutional | NA | Oral Cavity, Oropharynx, Larynx, Hypopharynx | 26; 18 with HPV-negative SCC, 7 with HPV-positive SCC | 66‡ | Not specified (pre-treatment) | ctDNA variant allele frequency (*ALK, ATM, BRCA1, BRCA2, CCND1, CDH1, CDK12, CDK2A, CHEK2, ERBB2, FGFR2, FGFR3, HRAS, NF1, PALB2,* and *PIK3CA*) | NGS  (pre-treatment) | Targeted NGS using FoundationOne^®^ Liquid (Foundation Medicine) | NA |
| Singh et al. 2024^43^ | To measure the levels of cfDNA in patients with HNSCC and to determine their possible association with the stage of the disease. | India | Prospective cross-sectional; single-institutional | NA | Oral Cavity, Oropharynx, Other Sites | 35 | 45‡ | Not specified (pre-treatment) | ctDNA detection levels | Spectrophotometry  (pre-treatment) | Ultraviolet spectrophotometry | Sensitivity: 43%, Specificity: 71% |
| Taylor et al. 2023^44^ | To characterize ctDNA dynamics under the treatment selection pressure of systemic therapy, correlating with clinical outcome in R/M HNSCC patients. | Canada | Prospective; single-institutional | 8.2 | Oral Cavity, Oropharynx, Larynx, Hypopharynx, Nasal Cavity | 53; 37 with HPV-negative SCC, 15 with HPV-positive SCC | 62 | Chemotherapy or immunotherapy | ctDNA variant allele frequency | NGS  (pre- and post-treatment) | CAPP-seq | NA |
| van Ginkel et al. 2017^45^ | To investigate whether low level ctDNA in plasma of patients with HNC can be detected using ddPCR. | The Netherlands | Retrospective; single-institutional | NA | Oropharynx | 6; All HPV-negative SCC | 61 | Surgery with or without chemotherapy/radiation therapy | ctDNA mutations (*TP53*) | NGS; PCR  (pre-treatment) | Targeted NGS using Ion Torrent Platform (Thermo Fisher Scientific); QX200 ddPCR system (Bio-Rad Laboratories). | NA |
| Verma et al. 2020^46^ | To investigate the role of ctDNA as a diagnostic marker in LA HNSCC and in monitoring response to chemoradiation therapy. | India | Prospective; single-institutional | 3 | Oral Cavity, Oropharynx, Larynx | 27 | NA | Chemoradiation therapy | ctDNA detection levels | PCR  (pre-and post-treatment) | qPCR: SYBR Green real-time PCR | Sensitivity: 100%, Specificity: 100% |
| Wilson et al. 2021^47^ | To characterize the genomic landscape of ctDNA in HNSCC and analyze its prognostic significance to precision oncology treatment alone and in combination with tumor DNA and to analyze concordance between tDNA and ctDNA sequencing. | USA | Retrospective; single-institutional | 8 | Oral Cavity, Oropharynx, Larynx, Hypopharynx, Nasopharynx, Sino-Nasal | 75; 33 with HPV-negative SCC, 20 with HPV-positive SCC | 60 | Surgery, radiotherapy, and/or chemotherapy | ctDNA mutations (including *TP53,*  *CDKN2A, TERT, BRCA2*, and *NOTCH1*) | NGS  (pre-and post-treatment) | Targeted NGS (FoundationOne, 323-gene tumor panel; Guardant360, 73-gene ctDNA panel) | Sensitivity: 38%, Specificity: 98% |

^‡^ Mean age is reported instead of median.

**Abbreviations**: HNSCC: head and neck squamous cell carcinoma; OSCC: Oral Cavity Squamous cell carcinoma; OPSCC: Oropharyngeal squamous cell carcinoma; LA HNSCC: Locoregionally advanced HNSCC; R/M HNSCC: recurrent/metastatic HNSCC; CUP: Cancer of Unknown Primary; MRD: minimal residual disease; NOS: not otherwise specified; ctDNA: circulating tumor DNA; cfDNA: cell-free DNA; tDNA: tumor DNA; HPV: Human Papillomavirus; NGS: Next-Generation Sequencing; PCR: Polymerase Chain Reaction; CAPP-seq: CAncer Personalized Profiling by deep sequencing; cfMeDIP-seq: cell-free Methylated DNA Immuno-Precipitation and high-throughput sequencing; SiMSen-Seq**:** Sensitive mutation detection using Sequencing; SafeSeq: Safe Sequencing System; cfMeDIP-seq: cell-free Methylated DNA Immuno-Precipitation and high-throughput sequencing; NextSeq: Next Sequencing; HiSeq: High Sequencing; ddPCR: Droplet Digital PCR; qMSP-ddPCR: quantitative methylation specific PCR; WGS: whole genome sequencing; USA: United States of America.

**References**

1. Burcher KM, Faucheux AT, Lantz JW, et al. Prevalence of dna repair gene mutations in blood and tumor tissue and impact on prognosis and treatment in hnscc. Article. *Cancers*. 2021;13(13)doi:10.3390/cancers13133118

2. Burgener JM, Zou J, Zhao Z, et al. Tumor-Naïve Multimodal Profiling of Circulating Tumor DNA in Head and Neck Squamous Cell Carcinoma. *Clin Cancer Res*. Aug 1 2021;27(15):4230-4244. doi:10.1158/1078-0432.Ccr-21-0110

3. Chikuie N, Urabe Y, Ueda T, et al. Utility of plasma circulating tumor DNA and tumor DNA profiles in head and neck squamous cell carcinoma. *Sci Rep*. Jun 4 2022;12(1):9316. doi:10.1038/s41598-022-13417-5

4. Cui Y, Kim HS, Cho ES, et al. Longitudinal detection of somatic mutations in saliva and plasma for the surveillance of oral squamous cell carcinomas. *PLoS One*. 2021;16(9):e0256979. doi:10.1371/journal.pone.0256979

5. de Jesus LM, Dos Reis MB, Carvalho RS, et al. Feasibility of methylated ctDNA detection in plasma samples of oropharyngeal squamous cell carcinoma patients. *Head Neck*. Nov 2020;42(11):3307-3315. doi:10.1002/hed.26385

6. de Vos L, Gevensleben H, Schröck A, et al. Comparison of quantification algorithms for circulating cell-free DNA methylation biomarkers in blood plasma from cancer patients. *Clin Epigenetics*. 2017;9:125. doi:10.1186/s13148-017-0425-4

7. Dietrich D, Weider S, de Vos L, et al. Circulating Cell-Free SEPT9 DNA Methylation in Blood Is a Biomarker for Minimal Residual Disease Detection in Head and Neck Squamous Cell Carcinoma Patients. *Clin Chem*. Sep 1 2023;69(9):1050-1061. doi:10.1093/clinchem/hvad084

8. Economopoulou P, Spathis A, Kotsantis I, et al. Next-generation sequencing (NGS) profiling of matched tumor and circulating tumor DNA (ctDNA) in head and neck squamous cell carcinoma (HNSCC). *Oral Oncol*. Apr 2023;139:106358. doi:10.1016/j.oraloncology.2023.106358

9. Egyud M, Sridhar P, Devaiah A, et al. Plasma circulating tumor DNA as a potential tool for disease monitoring in head and neck cancer. *Head Neck*. May 2019;41(5):1351-1358. doi:10.1002/hed.25563

10. Flach S, Howarth K, Hackinger S, et al. Liquid BIOpsy for MiNimal RESidual DiSease Detection in Head and Neck Squamous Cell Carcinoma (LIONESS)—a personalised circulating tumour DNA analysis in head and neck squamous cell carcinoma. Article. *British Journal of Cancer*. 2022;126(8):1186-1195. doi:10.1038/s41416-022-01716-7

11. Galot R, van Marcke C, Helaers R, et al. Liquid biopsy for mutational profiling of locoregional recurrent and/or metastatic head and neck squamous cell carcinoma. Article. *Oral Oncology*. 2020;104doi:10.1016/j.oraloncology.2020.104631

12. Grossi I, Assoni C, Lorini L, et al. Evaluation of DNA methylation levels of SEPT9 and SHOX2 in plasma of patients with head and neck squamous cell carcinoma using droplet digital PCR. *Oncol Rep*. Mar 2024;51(3)doi:10.3892/or.2024.8711

13. Hanna GJ, Dennis MJ, Scarfo N, et al. Personalized ctDNA for Monitoring Disease Status in Head and Neck Squamous Cell Carcinoma. *Clinical Cancer Research*. 30(15):3329-3336. doi:<https://dx.doi.org/10.1158/1078-0432.CCR-24-0590>

14. Hilke FJ, Muyas F, Admard J, et al. Dynamics of cell-free tumour DNA correlate with treatment response of head and neck cancer patients receiving radiochemotherapy. *Radiother Oncol*. Oct 2020;151:182-189. doi:10.1016/j.radonc.2020.07.027

15. Honoré N, van Marcke C, Galot R, et al. Tumor-agnostic plasma assay for circulating tumor DNA detects minimal residual disease and predicts outcome in locally advanced squamous cell carcinoma of the head and neck. *Ann Oncol*. Dec 2023;34(12):1175-1186. doi:10.1016/j.annonc.2023.09.3102

16. Honoré N, van der Elst A, Dietz A, et al. Tumour-agnostic plasma assay for circulating tumour DNA predicts outcome in recurrent and/or metastatic squamous cell carcinoma of the head and neck treated with a PD-1 inhibitor. *Eur J Cancer*. Dec 2023;195:113372. doi:10.1016/j.ejca.2023.113372

17. Huang Q, Ji M, Li F, et al. Diagnostic and prognostic value of plasma cell-free DNA combined with VEGF-C in laryngeal squamous cell carcinoma. *Mol Cell Probes*. Feb 2023;67:101895. doi:10.1016/j.mcp.2023.101895

18. Husain A, Singhal A, Agarwal A, Hadi R, Husain N. The role of circulating tumour dna as a marker of adequate resection in oral cancer and its correlation with clinicopathologic parameters: A prospective study. Article. *Journal of Clinical and Diagnostic Research*. 2020;14(3):XC16-XC19. doi:10.7860/JCDR/2020/43089.13593

19. Janke F, Stritzke F, Dvornikovich K, et al. Early circulating tumor DNA changes predict outcomes in head and neck cancer patients under re-radiotherapy. *Int J Cancer*. Aug 30 2024;doi:10.1002/ijc.35152

20. Kakimoto Y, Yamamoto N, Shibahara T. Microsatellite analysis of serum DNA in patients with oral squamous cell carcinoma. *Oncol Rep*. Nov 2008;20(5):1195-200.

21. Kampel L, Feldstein S, Tsuriel S, et al. Mutated TP53 in Circulating Tumor DNA as a Risk Level Biomarker in Head and Neck Squamous Cell Carcinoma Patients. *Biomolecules*. 2023;13(9):20. doi:<https://dx.doi.org/10.3390/biom13091418>

22. Khandelwal AR, Greer AH, Hamiter M, et al. Comparing cell-free circulating tumor DNA mutational profiles of disease-free and nonresponders patients with oropharyngeal squamous cell carcinoma. *Laryngoscope Investig Otolaryngol*. Oct 2020;5(5):868-878. doi:10.1002/lio2.447

23. Kogo R, Manako T, Iwaya T, et al. Individualized circulating tumor DNA monitoring in head and neck squamous cell carcinoma. *Cancer Med*. Nov 2022;11(21):3960-3968. doi:10.1002/cam4.4726

24. Koukourakis MI, Xanthopoulou E, Koukourakis IM, et al. Next-Generation Sequencing Analysis of Mutations in Circulating Tumor DNA from the Plasma of Patients with Head–Neck Cancer Undergoing Chemo-Radiotherapy Using a Pan-Cancer Cell-Free Assay. Article. *Current Oncology*. 2023;30(10):8902-8915. doi:10.3390/curroncol30100643

25. Koukourakis MI, Xanthopoulou E, Koukourakis IM, et al. Circulating Plasma Cell-free DNA (cfDNA) as a Predictive Biomarker for Radiotherapy: Results from a Prospective Trial in Head and Neck Cancer. *Cancer Diagn Progn*. Sep-Oct 2023;3(5):551-557. doi:10.21873/cdp.10254

26. Kumari S, Mishra S, Husain N, et al. Comparison of circulating DNA in malignant neoplasia from diverse locations: Investigating a diagnostic role. *Indian J Pathol Microbiol*. Jan-Mar 2022;65(1):93-99. doi:10.4103/ijpm.Ijpm_474_20

27. Kumari S, Mishra S, Anand N, Hadi R, Rastogi M, Husain N. Circulating free DNA integrity index and promoter methylation of tumor suppressor gene P16, DAPK and RASSF1A as a biomarker for oropharyngeal squamous cell carcinoma. *Pathol Res Pract*. Jun 2023;246:154489. doi:10.1016/j.prp.2023.154489

28. Lele SJ, Adilbay D, Lewis E, Pang J, Asarkar AA, Nathan CO. ctDNA as an Adjunct to Posttreatment PET for Head and Neck Cancer Recurrence Risk Assessment. *Otolaryngol Head Neck Surg*. Aug 2024;171(2):439-444. doi:10.1002/ohn.760

29. Lin LH, Chang KW, Kao SY, Cheng HW, Liu CJ. Increased Plasma Circulating Cell-Free DNA Could Be a Potential Marker for Oral Cancer. *Int J Mol Sci*. Oct 24 2018;19(11)doi:10.3390/ijms19113303

30. McKelvey BA, Andrews HS, Baehner FL, et al. Advancing Evidence Generation for Circulating Tumor DNA: Lessons Learned from A Multi-Assay Study of Baseline Circulating Tumor DNA Levels across Cancer Types and Stages. *Diagnostics*. 2024;14(9):27. doi:<https://dx.doi.org/10.3390/diagnostics14090912>

31. Mydlarz WK, Hennessey PT, Wang H, Carvalho AL, Califano JA. Serum biomarkers for detection of head and neck squamous cell carcinoma. *Head Neck*. Jan 2016;38(1):9-14. doi:10.1002/hed.23842

32. Nakagaki T, Tamura M, Kobashi K, et al. Targeted next-generation sequencing of 50 cancer-related genes in Japanese patients with oral squamous cell carcinoma. Article. *Tumor Biology*. 2018;40(9)doi:10.1177/1010428318800180

33. Nunes DN, Kowalski LP, Simpson AJ. Circulating tumor-derived DNA may permit the early diagnosis of head and neck squamous cell carcinomas. *Int J Cancer*. Apr 15 2001;92(2):214-9. doi:10.1002/1097-0215(200102)9999:9999<::aid-ijc1176>3.0.co;2-c

34. Oliva M, Chepeha D, Araujo DV, et al. Antitumor immune effects of preoperative sitravatinib and nivolumab in oral cavity cancer: SNOW window-of-opportunity study. Article. *Journal for ImmunoTherapy of Cancer*. 2021;9(10)doi:10.1136/jitc-2021-003476

35. Payne KFB, Brotherwood P, Suriyanarayanan H, et al. Circulating tumour DNA detects somatic variants contributing to spatial and temporal intra-tumoural heterogeneity in head and neck squamous cell carcinoma. *Front Oncol*. 2024;14:1374816. doi:10.3389/fonc.2024.1374816

36. Perdomo S, Avogbe PH, Foll M, et al. Circulating tumor DNA detection in head and neck cancer: Evaluation of two different detection approaches. Article. *Oncotarget*. 2017;8(42):72621-72632. doi:10.18632/ONCOTARGET.20004

37. Porter A, Natsuhara M, Daniels GA, et al. Next generation sequencing of cell free circulating tumor DNA in blood samples of recurrent and metastatic head and neck cancer patients. *Transl Cancer Res*. Jan 2020;9(1):203-209. doi:10.21037/tcr.2019.12.70

38. Sanz-Garcia E, Zou J, Avery L, et al. Multimodal detection of molecular residual disease in high-risk locally advanced squamous cell carcinoma of the head and neck. *Cell Death Differ*. Apr 2024;31(4):460-468. doi:10.1038/s41418-024-01272-y

39. Schröck A, Leisse A, de Vos L, et al. Free-Circulating Methylated DNA in Blood for Diagnosis, Staging, Prognosis, and Monitoring of Head and Neck Squamous Cell Carcinoma Patients: An Observational Prospective Cohort Study. *Clin Chem*. Jul 2017;63(7):1288-1296. doi:10.1373/clinchem.2016.270207

40. Schwaederle M, Chattopadhyay R, Kato S, et al. Genomic Alterations in Circulating Tumor DNA from Diverse Cancer Patients Identified by Next-Generation Sequencing. *Cancer Res*. Oct 1 2017;77(19):5419-5427. doi:10.1158/0008-5472.Can-17-0885

41. Shukla D, Kale AD, Hallikerimath S, Yerramalla V, Subbiah V. Can quantifying free-circulating DNA be a diagnostic and prognostic marker in oral epithelial dysplasia and oral squamous cell carcinoma? *J Oral Maxillofac Surg*. Feb 2013;71(2):414-8. doi:10.1016/j.joms.2012.04.039

42. Silvoniemi A, Laine J, Aro K, et al. Circulating Tumor DNA in Head and Neck Squamous Cell Carcinoma: Association with Metabolic Tumor Burden Determined with FDG-PET/CT. *Cancers*. 2023;15(15):04. doi:<https://dx.doi.org/10.3390/cancers15153970>

43. Singh S, Goyal R, Gupta A, et al. Role of Cell-free DNA as a Non-Invasive Biomarker in the Detection of Head and Neck Squamous Cell Carcinoma. *Indian journal of clinical biochemistry*. 2024;doi:10.1007/s12291-024-01181-4

44. Taylor K, Zou J, Burgener J, et al. Circulating tumor DNA kinetics in recurrent/metastatic head & neck squamous cell cancer (R/M HNSCC) patients. Conference Abstract. *Annals of Oncology*. 2021;32:S796-S797. doi:10.1016/j.annonc.2021.08.1296

45. van Ginkel JH, Huibers MMH, van Es RJJ, de Bree R, Willems SM. Droplet digital PCR for detection and quantification of circulating tumor DNA in plasma of head and neck cancer patients. *BMC Cancer*. Jun 19 2017;17(1):428. doi:10.1186/s12885-017-3424-0

46. Verma T, Kumari S, Mishra S, et al. Circulating free DNA as a marker of response to chemoradiation in locally advanced head and neck squamous cell carcinoma. *Indian J Pathol Microbiol*. Oct-Dec 2020;63(4):521-526. doi:10.4103/ijpm.Ijpm_28_20

47. Wilson HL, D'Agostino RB, Jr., Meegalla N, et al. The Prognostic and Therapeutic Value of the Mutational Profile of Blood and Tumor Tissue in Head and Neck Squamous Cell Carcinoma. *Oncologist*. Feb 2021;26(2):e279-e289. doi:10.1002/onco.13573
