# Supplementary material for: Circulating tumor DNA in Non-Viral head and neck squamous cell Carcinoma: A systematic review and Meta-Analysis
Source: Oral Oncol. Author manuscript; Available in PMC 2026 Jun 25. (PMC13299305; doi:10.1016/j.oraloncology.2025.107760)
Supplement: 4 [file NIHMS2186261-supplement-4.docx]

**Supplement 4.** Newcastle-Ottawa Scale (NOS) for assessment of quality of non-randomized trials.

| Study | Selection | Comparability | Outcomes | NOS Score |
| --- | --- | --- | --- | --- |
| Burcher et al. 2021 | ★★★ | ★★ | ★★★ | 8 |
| Burgener et al. 2021 | ★★★★ | ★★ | ★★★ | 9 |
| Chikuie et al. 2022 | ★★★ | ★ | ★★★ | 7 |
| Cui et al. 2021 | ★★★ | ★ | ★★★ | 7 |
| de Jesus et al. 2020 | ★★★★ | ★ | ★★★ | 8 |
| de Vos et al. 2017 | ★★★★ | ★ | ★★★ | 8 |
| Dietrich et al. 2023 | ★★★ | ★ | ★★★ | 7 |
| Economopoulou et al. 2023 | ★★★ | ★ | ★★★ | 7 |
| Egyud et al. 2019 | ★★★ |  | ★★★ | 6 |
| Flach et al. 2022 | ★★★ | ★ | ★★★ | 7 |
| Galot et al. 2020 | ★★★ | ★ | ★★★ | 7 |
| Grossi et al. 2024 | ★★★ | ★ | ★★★ | 7 |
| Hanna et al. 2024 | ★★★ | ★ | ★★★ | 7 |
| Hilke et al. 2020 | ★★★★ | ★★ | ★★★ | 9 |
| Honoré et al. 2023 | ★★★ | ★ | ★★★ | 7 |
| Honoré et al. 2023 (2) | ★★★ | ★★ | ★★★ | 8 |
| Huang et al. 2023 | ★★★★ | ★ | ★★★ | 8 |
| Husain et al. 2020 | ★★★ | ★ | ★★★ | 7 |
| Janke et al. 2024 | ★★★ | ★ | ★★★ | 7 |
| Kakimoto et al. 2008 | ★★★ |  | ★★★ | 6 |
| Kampel et al. 2023 | ★★★ | ★ | ★★★ | 7 |
| Khandelwal et al. 2020 | ★★★ | ★ | ★★★ | 7 |
| Kogo et al. 2022 | ★★★ | ★ | ★★★ | 7 |
| Koukourakis et al. 2023 | ★★★ | ★ | ★★★ | 7 |
| Koukourakis et al. 2023 (2) | ★★★ | ★ | ★★★ | 7 |
| Kumari et al. 2022 | ★★★ | ★ | ★★ | 6 |
| Kumari et al. 2023 | ★★★★ | ★ | ★ | 6 |
| Lele et al. 2024 | ★★★ | ★ | ★★★ | 7 |
| Lin et al. 2018 | ★★★★ | ★ | ★★★ | 8 |
| McKelvey et al. 2024 | ★★★ | ★ | ★★ | 6 |
| Mydlarz et al. 2016 | ★★★★ | ★★ | ★ | 7 |
| Nakagaki et al. 2018 | ★★★ | ★★ | ★ | 6 |
| Nunes et al. 2001 | ★★★★ | ★ | ★ | 6 |
| Oliva et al. 2021 | ★★★ | ★ | ★★★ | 7 |
| Payne et al. 2024 | ★★★ | ★ | ★★★ | 7 |
| Perdomo et al. 2017 | ★★★★ | ★ | ★ | 6 |
| Porter et al. 2020 | ★★★ | ★ | ★★ | 6 |
| Sanz-Garcia et al. 2024 | ★★★ | ★ | ★★★ | 7 |
| Schrock et al. 2017 | ★★★★ | ★★ | ★★★ | 9 |
| Schwaederle et al. 2017 | ★★★ | ★ | ★★ | 6 |
| Shukla et al. 2013 | ★★★★ | ★ | ★ | 6 |
| Silvoniemi et al. 2023 | ★★★ | ★ | ★★ | 6 |
| Singh et al. 2024 | ★★★★ | ★ | ★ | 6 |
| Taylor et al. 2023 | ★★★ | ★★ | ★★★ | 8 |
| van Ginkel et al. 2017 | ★★★ | ★ | ★★ | 6 |
| Verma et al. 2020 | ★★★★ | ★ | ★★★ | 8 |
| Wilson et al. 2021 | ★★★ | ★★ | ★★★ | 8 |
