# Supplementary material for: Circulating tumor DNA in Non-Viral head and neck squamous cell Carcinoma: A systematic review and Meta-Analysis
Source: Oral Oncol. Author manuscript; Available in PMC 2026 Jun 25. (PMC13299305; doi:10.1016/j.oraloncology.2025.107760)
Supplement: 5 [file NIHMS2186261-supplement-5.docx]

**Supplement 5.** Comparison of ctDNA detection methods: advantages and limitations.

| **Method** | **Examples** | **Advantages** | **Limitations** |
| --- | --- | --- | --- |
| **ddPCR** | Bio-Rad QX200 | Very high sensitivity for specific mutations; precise absolute quantitation; low cost per assay | Limited to small number of known loci; needs prior knowledge of mutation |
| **qPCR** | SYBR Green real-time PCR, CF X96 Real-Time PCR system | Fast, widely available, quantitative; can detect known mutations; relatively low cost | Limited multiplexing; lower sensitivity than ddPCR for very low allele frequencies; requires prior knowledge of target |
| **Next-Generation Sequencing** | Illumina NextSeq, Ion Torrent, Guardant360 | Can detect multiple mutations simultaneously; broad genomic profiling; can track tumor evolution; supports mutation and CNV | Higher cost; requires moderate DNA input; lower sensitivity for very low VAF without UMIs/deep sequencing |
| **Whole-exome/Whole-genome sequencing** | Illumina NovaSeq, HiSeq | Comprehensive genomic coverage; can discover novel variants; supports research and tumor evolution studies | Very high cost; requires high DNA input; not optimal for ultra-sensitive MRD; slower turnaround |
| **Fluorometry** | Qubit dsDNA Assay (Thermo Fisher) | Quantification of total cell-free DNA; fast and simple; low cost | Cannot detect mutations; no genomic information |
| **Spectrophotometry** | NanoDrop, Thermo Fisher | Quick, simple, and inexpensive; measures total DNA | Low sensitivity; cannot distinguish cfDNA from contaminating DNA; not suitable for mutation detection |

**Abbreviations:** ddPCR: droplet digital Polymerase Chain Reaction; qPCR: quantitative Polymerase Chain Reaction; VAF: Variant Allele Frequency; UMIs: Unique Molecular Identifiers; MRD: Minimal Residual Disease; cfDNA: cell-free Tumor DNA; CNV: Copy Number Variation.
